# ﻿The definitive rediscovery of *Telmatobiushalli* (Anura, Telmatobiidae) at its historic type locality and its synonymy with *T.dankoi* and *T.vilamensis*

**DOI:** 10.3897/zookeys.1079.69036

**Published:** 2021-12-22

**Authors:** Jakob von Tschirnhaus, Claudio Correa

**Affiliations:** 1 Lamark 77, Valparaíso, Chile Unaffiliated Valparaíso Chile; 2 Laboratorio de Sistemática y Conservación de Herpetozoos, Departamento de Zoología, Facultad de Ciencias Naturales y Oceanográficas, Universidad de Concepción, Barrio Universitario S/N, Concepción, Chile Universidad de Concepción Concepción Chile

**Keywords:** Amphibia, Chile, Loa River, lost frog, phylogeny, Puna, taxonomy

## Abstract

*Telmatobiushalli* was the first representative of its genus to be described exclusively for Chile, yet for 85 years no new individuals could be located due to the vagueness with which its type locality was described. The type series was collected by one of the members of the International High Altitude Expedition to Chile (IHAEC) of 1935. Recently, three studies successively claimed to have located the type locality in different places. The third study proved, according to the chronicles of the IHAEC, that the actual locality is Miño, at the origin of the Loa River, where currently there are no published records of *Telmatobius*. In this study, additional documentary antecedents and graphic material are provided that corroborate that Miño is indeed the type locality of *T.halli.* Additionally, the recently rediscovered *Telmatobius* population from Miño and the environment it inhabits are described. The external characteristics of the frogs are consistent with the description of *T.halli*. Furthermore, molecular phylogenetic analyses were performed that showed that *T.halli*, *T.dankoi*, and *T.vilamensis*, all known only from their type localities in Chile, comprise a clade without internal resolution. A detailed comparison of the diagnoses of the three species revealed that the few phenotypic differences between these taxa were based on characteristics that vary widely within and between populations of the genus, hence their conspecificity is proposed. The implications of this synonymy for the taxonomy, biogeography, and conservation of the *Telmatobius* from the extreme south of its distribution in Chile are discussed.

## ﻿Introduction

The genus *Telmatobius* Wiegmann, 1834 is one of the few anuran taxa that has managed to diversify in the high Andes (Barrionuevo 2017), so its representatives exhibit a series of physical and physiological adaptions that allow them to survive in such harsh environments (e.g., Ruiz et al. 1983; Reider et al. 2020). In fact, it includes one of the highest-dwelling frog species, *Telmatobiusmarmoratus* (Duméril & Bibron, 1841), which has been reported from up to 5,400 m (Reider et al. 2020). Currently, there are 63 recognized species in this genus (Frost 2021), dispersed throughout a variety of ecosystems alongside the Andes, between approximately 1°S and 30°S (Barrionuevo 2017). The biogeographical consequences of the uplifting of the Andes during the late Pliocene and Pleistocene and paleoclimatic processes, such as the repeated formation and evaporation of extensive lakes, have been proposed to be responsible for the diversification of the fauna in the Puna highlands, the driest section of the Central Andes (e.g., Collado et al. 2011; Vila et al. 2013; Sáez et al. 2014). Thus, vicariance seems to be a reasonable explanation for the divergence of the *Telmatobius* from this arid region, taking into account their strongly aquatic habits (Barrionuevo 2017) and the hostile environments that have evolved around the watercourses.

In Chile, nine species of *Telmatobius*, seven of them endemic, are currently recognized (Fabres et al. 2018; Correa 2019), although Sáez et al. (2014) questioned the presence of *Telmatobiusperuvianus* Wiegmann, 1834 in Chilean territory. Furthermore, Sáez et al. (2014) suggested that *Telmatobiusdankoi* Formas, Northland, Capetillo, Nuñez, Cuevas & Brieva, 1999 and *Telmatobiusvilamensis* Formas, Benavides & Cuevas, 2003 might be conspecific (see also Fabres et al. 2018) and pointed out the low genetic divergence between *Telmatobiusphilippii* Cuevas & Formas, 2002 and *Telmatobiusfronteriensis* Benavides, Ortiz & Formas, 2002. Despite these taxonomic uncertainties, the number of known populations of the genus in Chile has increased substantially over the last decade (e.g. Sáez et al. 2014; Victoriano et al. 2015; Fibla et al. 2017; Lobos and Rojas 2020). Undoubtedly, a greater sampling effort will expose even more discoveries, yet the accessibility and the conditions for fieldwork in the region are challenging.

As in the case of other Chilean amphibian genera (*Alsodes* Bell, 1843; *Eupsophus* Fitzinger, 1843) (Blotto et al. 2013; Correa and Durán 2019), the taxonomy of the genus *Telmatobius* is complex due to high levels of intra- and interpopulation variation in morphological features (e.g., Trueb 1979; Wiens 1993; De la Riva et al. 2005; Barrionuevo 2017), especially in characters that are traditionally used to distinguish anuran species (De la Riva et al. 2005). Thus, molecular studies have played an important role in the systematics of this taxonomic group in Chile during the past few years (Sáez et al. 2014; Victoriano et al. 2015; Fibla et al. 2017, 2018 – reviewed by Sáez and Méndez 2020).

**Figure 1.**
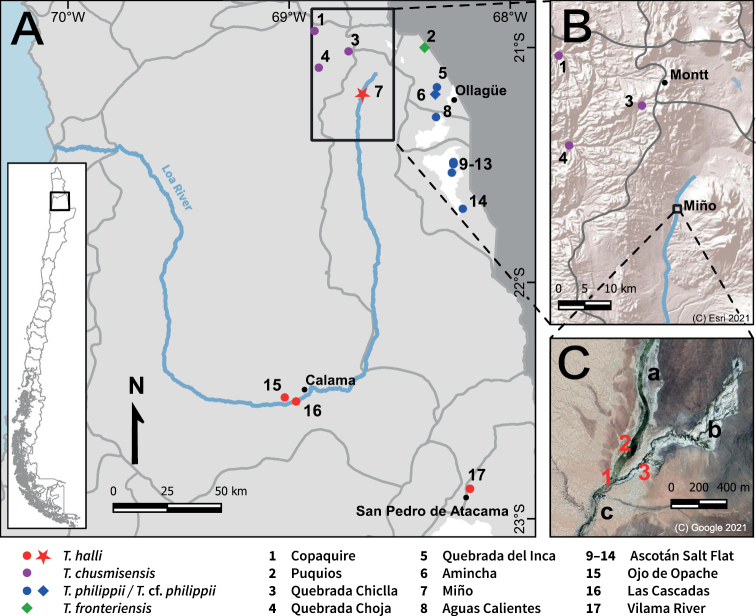
Geographic context of this study **A** distribution of all *Telmatobius* populations known from the southern range of the genus in Chile (20°55'–22°55'S). Light grey area = Chilean territory, dark grey area = Bolivian territory, grey lines = limits of the sub-basins, star = study site, diamonds = type localities **B** topographic relief of the surroundings of Miño. Grey lines = limits of the sub-basins. Montt is the name provided by the IHAEC for the Collahuasi Copper Mine **C** satellite image of Miño. 1) Concrete pool, 2) ruins of mining settlement, 3) sampling point, a) Miño River, b) Nacimiento Creek, c) Loa River.

Among the endemic species of Chile, *Telmatobiushalli* Noble, 1938 stands out for its complex taxonomic history. Dr Frank Gregory Hall collected the type series (adults and larvae) in the context of the International High Altitude Expedition to Chile (**IHAEC**), an endeavor that took place in 1935 and whose principal purpose was to study the effects of low-oxygen environments of high elevation on the human physiology and the body’s acclimatization response (Keys 1936b). Three years after the expedition, Dr Gladwyn Kingsley Noble, from the American Museum of Natural History (AMNH), described the specimens he had received from Chile and named the species after its collector. There has been considerable confusion regarding *T.halli* and most part of it must be ascribed to Noble’s vague definition of the type locality as “Warm spring near Ollagüe, Chile, 10,000 ft. altitude” (Correa 2021). Throughout the years, a few populations had been assumed to belong to *T.halli* (Capurro 1954, 1955; Cei 1962, 1986; Veloso et al. 1982; Northland et al. 1990; Núñez and Gálvez 2015), but were later revised and described as new species (*T.dankoi*Formas et al., 1999; *T.vilamensis*Formas et al., 2003) or assigned to another taxon, like in the case of the populations from Ascotán Salt Flat, treated as T.cf.philippii by Lobos et al. (2018) (Fig. 1). Furthermore, anurans found at Tatio, San Pedro de Atacama were described as the subspecies *T.halliedentatus* (Capurro, 1955), but Cei (1962) identified the specimens in question as *Rhinellaspinulosa* (Wiegmann, 1834).

Formas et al. (2003) redescribed *T.halli* based on the type material from the AMNH and differentiated it from *T.dankoi* and *T.vilamensis* using morphological evidence. During the last three decades, significant efforts were made to locate the type locality of *T.halli* (Formas et al. 2003, 2005; IUCN 2015). These expeditions were infructuous in terms of clarifying the whereabouts of *T.halli*, but led to the description of new species (*Telmatobiusphilippii* Cuevas & Formas, 2002, *T.fronteriensis*Benavides et al., 2002) and the discovery of a series of undetermined populations (*Telmatobius* sp. from Ascotán and Carcote salt flats; Sáez et al. 2014) in the area surrounding Ollagüe (Fig. 1).

Recently, Fibla et al. (2018) and Cuevas et al. (2020) independently claimed to have rediscovered *T.halli*. Bibliographic sources describing the IHAEC’s activities were used in both studies, but each focused on different known populations of *Telmatobius*. Thus, Fibla et al. (2018) assigned the southernmost populations of *T.chusmisensis* Formas, Cuevas & Nuñez, 2006 (sensu Sáez et al. 2014) to *T.halli* (Copaquire, Quebrada Choja, Quebrada Chiclla), while Cuevas et al. (2020) did the same with a population from the Carcote Salt Flat (specifically, from the hot spring Aguas Calientes) (Fig. 1A, B). Previously, a Carcote population (coordinates not specified) was considered as *Telmatobius* sp. by Sáez et al. (2014) or T.cf.philippii by Lobos et al. (2018). According to the molecular phylogenetic analyses of Sáez et al. (2014), the only study where all of these populations were included, they are nested in different clades, the *T.pefauri* (former *T.zapahuirensis*, see Fibla et al. 2017) and *T.hintoni* species groups (not recovered in the most recent analysis by Barrionuevo 2017), respectively, so they clearly do not correspond to the same taxon. Nevertheless, the opposing hypotheses of Fibla et al. (2018) and Cuevas et al. (2020) were refuted by Correa (2021), who demonstrated, also using bibliographic sources, that the frog was first found near a warm concrete swimming pool in Miño, a location at the source of Loa River, at the western foot of Miño Volcano (Fig. 1C). In the literature, there are no other reports of *Telmatobius* populations neither from Miño nor from the upper Loa basin.

Altogether, 83 years after its description and despite the multiple recent hypotheses about the location of its type locality and identity, *T.halli* is still a lost frog and no specimens have ever been seen since the collection of the type series (Correa 2021). Hence, the main goal of this contribution is to describe the *Telmatobius* population found in Miño, the place recently identified as the true type locality of *T.halli* by Correa (2021). We provide a general description of the location, some observations on adults and tadpoles, and basic information on the quality of their habitat. We also provide additional documentary and graphic evidence that corroborate the results of Correa (2021). Furthermore, we performed phylogenetic analyses to shed light on the systematic relationships among the population of Miño, the ones recently proposed as being *T.halli* (Copaquire, Quebrada Choja, Quebrada Chiclla and Carcote Salt Flat) (Fibla et al. 2018; Cuevas et al. 2020) and other *Telmatobius* species, which, prior to their description, had been postulated to be *T.halli* (*T.vilamensis* and *T.dankoi*) (e.g., Cei 1962; Veloso et al. 1982). Finally, we compared the diagnoses of *T.halli* with those of the latter two species to re-evaluate their taxonomic status.

## ﻿Materials and methods

### ﻿Archival evidence

The diary of Ross McFarland, one of the members of the IHAEC, was requested from the Ross A. McFarland Collection in Aerospace Medicine and Human Factors Engineering at the Wright State University Archives. The diary is listed as “Box 63, Folder 5: Ross McFarland’s Diary (May 1935–September 1935)” in the collection’s inventory (Hoffman and Ritchie 1987: 29). From the same collection, we obtained the video footage recorded by McFarland during the expedition (Items 2213, 2217 and 2218; Hoffman 1987: 113), which shows a concrete swimming pool at the source of Loa River. Individual frames were extracted from the video and panoramic views of the four different positions of the cameraman were generated, using the open-source software HUGIN – Panorama photo stitcher (version 2019.2.0).

### ﻿Study area

On 31 October 2020, a field trip to the site called Miño (21°12'S, 68°40'W; 3900 m elevation; Calama Commune, El Loa Province, Antofagasta Region, Chile) was performed to locate the frog population that was described as *T.halli* (Correa 2021). The historical reference for this search was based on the swimming pool and other features of the landscape that appear in the recordings made by McFarland.

As biosecurity measures to prevent the spreading of chytridiomycosis and other infectious diseases, we disinfected car tires, boots, and utensils with F10 Super Concentrate Disinfectant (Health and Hygiene Pty.) at a concentration of 1:250 (Webb et al. 2007).

### ﻿Ecology

We made a general description of the study area, considering the topography of the landscape and more specific conditions at microhabitat level. We measured the stream dimensions at various points and took air and water temperatures at different times of the day. The composition of the adjacent vegetation along the stream was ascertained and a nocturnal survey was undertaken to detect possible sympatric amphibians.

Both, adults and larvae, identified as *Telmatobius*, were collected during the daytime from the stream using a hand net. The sampling site was ~ 300 m upstream from the pool identified as the historical place where *T.halli* was collected (see details in Results). The animals were measured, photographed, and finally released back to the capture site. Each individual was handled separately with an unused pair of disposable nitrile gloves (Thomas et al. 2020). To avoid possible toxic effects, the gloves were rinsed and the rinse water was discarded away from the watercourse (Cashins et al. 2008).

In order to obtain bioacoustic data, an AudioMoth recording unit (Hill et al. 2019) was placed beside the stream, at a spot where adult individuals had been sighted during sampling. The device recorded continuously between 8 p.m. and 7 a.m., but we did not obtain vocalizations that could be unquestionably attributed to *Telmatobius*.

During the night, the AudioMoth took a measurement of the air temperature every 15 minutes, but the sensor only has an accuracy of ± 3 °C (Open Acoustic Devices 2020). Water temperature was taken with a generic digital thermometer.

### ﻿Morphometrics

Seven morphometric features were measured on 11 adult specimens (Watters et al. 2016): snout-vent length (**SVL**), head width (**HW**), head length (**HL**), inter-orbital distance (**IOD**), inter-nostril distance (**IND**), foot length (**FL**) and tibia length (**TL**). FL and TL were assessed on the right hindlimb. In the case of the tadpoles (n = 9), body length (**BL**) and total length (**TTL**) were measured (Altig 2007) and the development stages (Gosner 1960) were determined. All measurements were taken using a vernier caliper to the nearest 0.05 mm.

### ﻿Sampling and obtaining DNA sequences

Three tadpoles (Gosner stages 36–37) were anesthetized by immersing them in a buffered solution of MS-222 (0.2%) (Mitchell 2009), and a small portion of the membrane was cut from the caudal fin. After recovery from the anesthesia, they were released at the collection site. The tissue samples were stored in 96% ethanol until DNA extraction.

The DNA was extracted with a commercial kit (Promega ReliaPrepTM gDNA Tissue Miniprep System, Madison, WI) following the manufacturer’s instructions. We obtained fragments of two mitochondrial genes, 16S rRNA and cytochrome b (cytb), the same fragments that were used in the phylogenetic analyses of Sáez et al. (2014). The reagent mixtures, reaction conditions, and primers used in the PCRs are detailed in Sáez et al. (2014) and references therein. Electropherograms were edited with the program Bioedit v7.1.3 (Hall 1999). Substitu­tion saturation of the sequences was assessed with DAMBE7 (Xia 2018). Sequences were deposited in GenBank (accession numbers OL412556–OL412561).

### ﻿Phylogenetic analyses

The sequences of both fragments were aligned with MUSCLE (Edgar 2004) and the alignments were then inspected by eye. Bayesian phylogenetic analyses were performed with the program MrBayes v3.2.7 (Ronquist et al. 2012), in which all *Telmatobius* species from Chile and all sampled populations of the genus geographically close to Miño were included (Appendix 1). Both gene fragments were concatenated, but a reversible-jump Markov Chain Monte Carlo method for exploring the space of all General Time Reversible sub-models, plus gamma and proportion of invariable sites parameters, was applied independently to each fragment (an additional analysis was carried out considering the different positions of the codons of the cytb as distinct partitions). Both analyses consisted of two groups of four Markov chains that run independently for 20 million generations, sampling every 1,000 gener­ations. The first 25% of generations was conservatively discarded as burn-in after observing the stationarity of ln-likelihoods of trees in Tracer v1.7.1 (Rambaut et al. 2018). Convergence and mixing of chains were assessed by examining values of average standard deviation of split frequencies (ASDSF) and expected sampling sizes (ESS) and Potential Scale Reduction Factor (PSRF) for all parameters. Sampled trees were rooted with one specimen of *Telmatobiussibiricus* De la Riva & Harvey, 2003, a representative of the *T.bolivianus* species group (Sáez et al. 2014; Barrionuevo 2017) which constitutes the sister clade of the three species groups present in Chile (Sáez et al. 2014).

### ﻿Comparison of the morphology of *T.halli*, *T.dankoi* and *T.vilamensis*

We collected all available information on the morphology of *T.halli* and the two populations to which the same name was assigned before being formally described as different species (*T.dankoi* and *T.vilamensis*) to compare their diagnostic characters as well as the proposed differences between them. The morphological details were obtained from the literature as follows: *T.halli* (Noble 1938; Veloso et al. 1982; Formas et al. 1999, 2003), *T.dankoi* (Veloso et al. 1982; Formas et al. 1999, 2003; Barrionuevo 2017), *T.vilamensis* (Benavides et al. 2002; Formas et al. 2003; Barrionuevo 2017). We further contrasted our observations of adults from Miño with the published data and added some minor comments regarding morphological traits observed in the populations from Las Cascadas (*T.dankoi*) and Vilama River (*T.vilamensis*).

## ﻿Results

### ﻿The type locality

**Figure 2.**
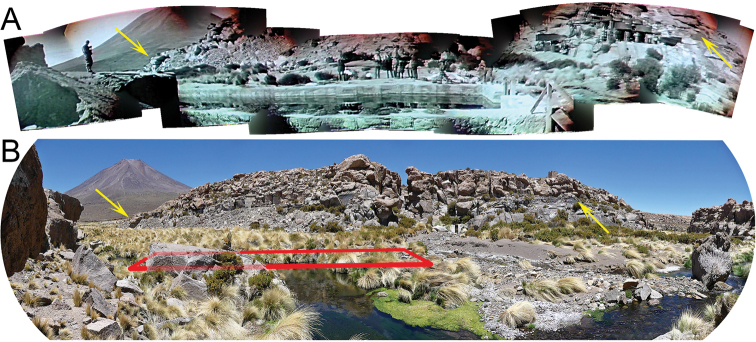
Historic and current panoramic view of the area surrounding the concrete swimming pool in Miño **A** panorama extracted from video footage from the IHAEC, 1935. Yellow arrows indicate rock formations that are easily recognizable **B** current state of the habitat. Red rectangle = location of the concrete pool. The mountain in the left background is Miño Volcano.

**Figure 3.**
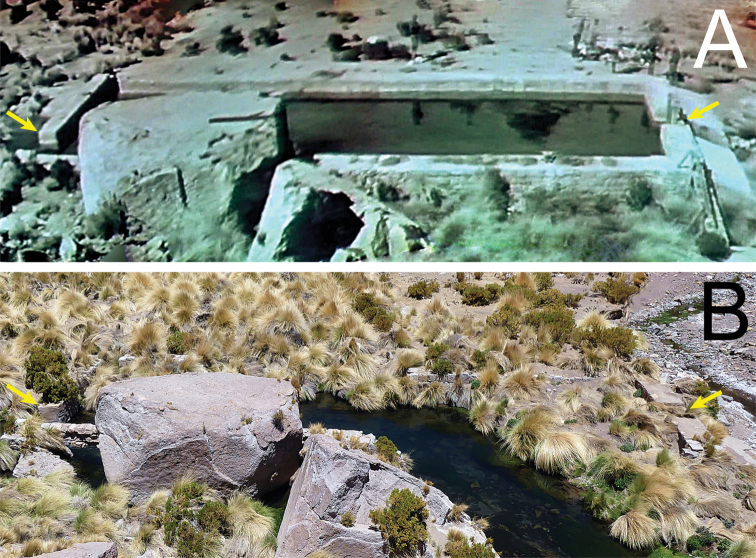
Historic and current view of the concrete swimming pool in Miño **A** panorama extracted from video footage from the IHAEC, 1935. Yellow arrows indicate the upper and lower pool walls **B** same view in 2020.

**Figure 4.**
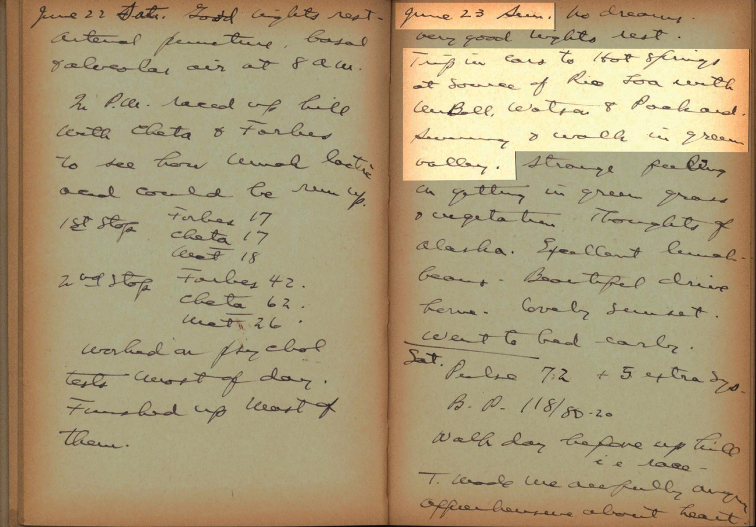
Extract from the diary Ross McFarland wrote in 1935 during the IHAEC.

As pointed out in Correa (2021), according to the chronicles of the IHAEC by Keys (1936a, b) and Dill (1979, 1980), the collection site of the type series of *T.halli* was the surroundings of a concrete swimming pool filled with warm water at the source of the Loa River (Figs 2, 3). Here, we provide the additional historical evidence extracted from the diary and the video recording of Ross McFarland that allowed us to identify the exact spot of the type locality. In the diary entry for Sunday, 23 June 1935, he wrote: “Trip in cars to hot springs at source of Rio Loa with Mr. Bell, Watson & Packard. Swimming & walk in green valley.” (McFarland 1935) (Fig. 4). The diary also confirms that the date of the departure of the IHAEC from Collahuasi (railway station Montt) (Fig. 1B) back to Ollagüe was Tuesday, 25 June 1935. As stated in Fibla et al. (2018), this means that the original collection date was June 23 and not June 25, as specified by Noble (1938).

Regarding McFarland’s video material (Suppl. material 1), the mountain in the background of the video takes can easily be identified, even using Google Earth’s perspective view, as Miño Volcano, because of a characteristic bulge in its profile. Although strong erosion events have reshaped part of the landmarks, multiple rock formations of the canyon walls still remain identical and corroborate the congruence of the place with respect to the one depicted in the video (Fig. 2).

As expected, the remains of the mentioned concrete swimming pool were found at 21°12'01"S, 68°40'09"W (3,900 m) (Fig. 3). Even though the stream broke through the lower end of the pool’s wall and the bottom is filled with sand, most of the boundaries are still in place and it is evident that the structure corresponds to the one shown in the recording. The pool is rectangular, approximately 6.5 m wide, 20 m long and between 1.5 and 2 m deep. The side walls are made of stones, joined together with concrete, while the upper and lower walls comprise massive concrete blocks. There are other more recent concrete structures inside the stream, one immediately above the pool and another one ~ 300 m upstream.

### ﻿Habitat description

The Loa River originates mainly from meltwater from throughout its upper drainage basin, where snow accumulates during austral winter. Several temporal ravines also gather the characteristic precipitations during the austral summer months (December to March), known as Altiplanic winter (Berenguer and Cáceres 2008; Delsouc et al. 2020). Lower down and descending from the east, there also are some important permanent affluents fed by aquifers.

For the first few kilometers, the riverbed is a broad and dry wadi named Miño River. Only ~ 4 km north of Miño, the arid riverbed gradually turns greener and ends in a small bog with grass tussocks, covering an area of ~ 5 ha. No significant water flow was registered during this time of the year (late October). At Miño, there are some well-preserved ruins of an old mining camp from the 18^th^ and 19^th^ centuries at both sides of the bog (Berenguer and Cáceres 2008), serving as an easily recognizable landmark (Fig. 1C).

From this point on, the river bears the name Loa, as it receives its first permanent tributary, the “Estero Nacimiento” creek (Berenguer and Cáceres 2008). This spring emerges at the head of a small ravine of ~ 1.3 km in length, a place called Ojos del Miño (21°11'43"S, 68°39'40"W) (Flores 2001) (Fig. 1C).

Below the confluence, the river suddenly turns into a pronounced canyon with vertical cliffs. The concrete pool is located precisely at the beginning of the canyon. Soon after, the river gets a little broader, forming larger natural ponds and sections with rapids. The canyon goes on in a similar manner for almost 100 km, until reaching the Conchi water reservoir.

**Table 1.** Currently known *Telmatobius* populations from the southern range of the genus in Chile (20°55'–22°55'S). Localities are ordered from north to south (Fig. 1). Bold letters denote type localities. Asterisk (*) indicates that the elevation was obtained from Google Earth (expressed in m a.s.l.). Note that the specific names assigned to Copaquire, Quebrada Chiclla, Quebrada Choja and Aguas Calientes populations correspond to the taxonomy prior to the proposals of Fibla et al. (2018) and Cuevas et al. (2020). The populations of Las Cascadas, Ojo de Opache and Vilama River are labeled according to the taxonomic changes proposed in this study.

Species	Locality	Elevation	References
*T.chusmisensis*	Copaquire	3,540*	Sáez et al. (2014)
*T.fronteriensis*	**Puquios**	4,150	Benavides et al. (2002)
*T.chusmisensis*	Quebrada Chiclla	4,550*	Sáez et al. (2014)
*T.chusmisensis*	Quebrada Choja	3,500*	Sáez et al. (2014)
*T.philippii*	Quebrada del Inca	3,800	Cuevas and Formas (2002)
*T.philippii*	**Quebrada de Amincha**	3,800	Cuevas and Formas (2002)
*T.halli*	**Miño**	3,900*	this study
Telmatobiuscf.philippii	Aguas Calientes^1^	3,717	Lobos et al. (2018), Cuevas et al. (2020)
Telmatobiuscf.philippii	Ascotán Salt Flat (springs 2, 3, 5, 6, 7 and 11)	~ 3,720	Lobos et al. (2018)
*T.halli* (previously *T.dankoi*)	Ojo de Opache (introduced there in 2019)	1,960*	Lobos and Rojas (2020)
*T.halli* (previously *T.dankoi*)	Las Cascadas	2,260	Formas et al. (1999)
*T.halli* (previously *T.vilamensis*)	Vilama River	2,250*	Formas et al. (2003)

^1^There are several publications prior to Cuevas et al. (2020) that include specimens of a population of *Telmatobius* sp. of the Carcote Salt Flat (Sáez et al. 2014; Fibla et al. 2017, 2018), but none of them specify the coordinates or a precise site within the salt flat. Lobos et al. (2018) mention the population of the Carcote Salt Flat (as Telmatobiuscf.philippii), but only in Lobos et al. (2020) are the coordinates specified (in Table S1 of their supplementary material), which fall very close to the Cuchicha spring (not shown in the map of Fig. 1A), located ~ 1.9 km NE of Aguas Calientes.

### ﻿Microhabitat and ecology

At the sampling point, the current of the Nacimiento Creek flows rapidly, though the terrain is not very steep. The stream is between 2.5 and 5 m broad and 25–50 cm deep. The water is clear and the bottom is mostly sandy with some stones and scarce detritus at the bends. The margins are almost entirely covered with vegetation, mainly *Festucachrysophylla* Phil. and a few bushes of *Parastrephialucida* (Meyen) Cabrera. The overhanging grass cushions are ideal refugia for the frogs, forming at times gallery-like cavities along the riverbank. At some points inside the stream, patches of *Myriophyllumaquaticum* (Vell.) Verdc. can be found, alternating with mats of undetermined filamentous green algae.

At the pool site, the bottom is also sandy; however, there is a little more mud and detritus, probably coming from the bog and consequently a more abounding aquatic vegetation. The stream at the exit of the pool measures ~ 4 m in width and 50 cm in depth. Downstream from the pool, the vegetation coverage at the banks decreases a bit, which leaves fewer shelters for the frogs. In fact, a lower population density was detected there.

Adults of *T.halli* were found mainly under the tussocks, where they shared their refugia with other adults and larvae. On one occasion, 11 adults and one tadpole were captured from below the same plant. Tadpoles also exhibit gregarious habits, but somehow seem to prefer to shelter inside the aquatic vegetation, at the bottom of the stream. Still, they are not absent under the cushions at the riverbank. Most of the observed larvae were at approximately the same development stage (Gosner stage 36–37); however, two specimens were younger (Gosner stages 27 and 33). Directly inside the pool, there were very few *Telmatobius* tadpoles and only one adult was found a few meters below the outlet.

During the daytime, two adults of *Rhinellaspinulosa* were found under the riparian vegetation in the pool and after nightfall, numerous individuals of these toads were observed outside the water along the stream. A small ravine, adjacent to the pool, was occupied by hundreds of *Rhinella* larvae in semi-lentic, shallow puddles, which are ideal for their development. Additionally, one specimen of *Pleurodemamarmoratum* (Duméril & Bibron, 1840) was found walking around at night; hence, all three potential anuran species were present in the area. Since no case of syntopy between the Chilean *Telmatobius* has been reported, no other congener is expected to be encountered in Miño.

### ﻿Temperature

In the afternoon (05:00 p.m.), the air temperature was 21.8 °C, almost equal to the water temperature at the outlet of the pool (21.4 °C). In contrast, in the morning (8:00 a.m.) the air temperature was -2.4 °C, while water temperatures at the pool and the sampling site were 19.0 °C and 20.7 °C, respectively. After sunset, the air temperature dropped quickly to around -11.0 °C (00:30 a.m.) and remained alike until dawn. The minimum value was -13.1 °C at 03:30 a.m. The water temperature, which is generally higher than that of other localities of the genus (Lobos and Rojas 2020) and which remains more or less constant (19–21.4 °C), is consistent with the description of the original capture site (“a warm spring”; Noble 1938).

### ﻿Morphology

**Figure 5.**
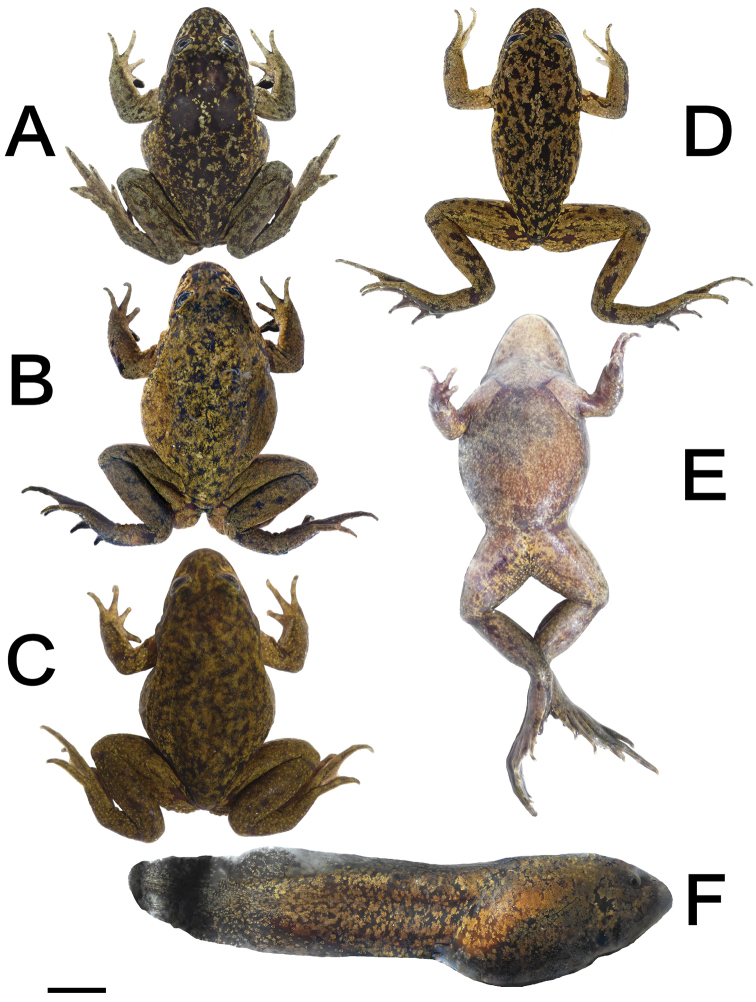
Selected specimens of *Telmatobiushalli* from Miño **A–D** dorsal views of adult specimens, showing variation in coloration patterns **E** ventral view of the specimen from C **F** tadpole; scale bar: 1 cm (**A–F**).

**Figure 6.**
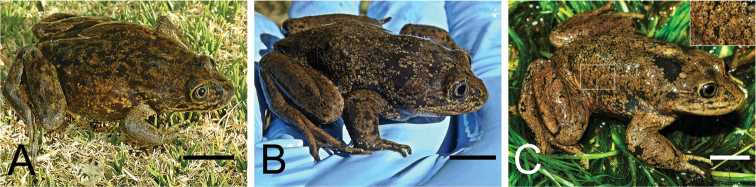
Adults from the three known populations of *Telmatobiushalli*, as recognized in this study, showing the similarity in their external appearance **A** Miño **B** Las Cascadas and **C** Vilama River. The inlay in the upper right corner of **C** shows a detail of the keratinous spines. Photograph credits for the Vilama River specimen: Felipe Rabanal. Scale bars: 1 cm.

Overall, *T.halli* is a medium-sized frog (Table 2), with a depressed body, thin forelimbs and anterodorsolaterally orientated eyes (Fig. 5). In dorsal view, the head is slightly broader than long (HL/HW = 0.96), but narrower than the body. On average the head length is 29.65% of SVL. The snout tends to be long but truncated in dorsal view, although it can be rather elliptical in some individuals. In lateral view, the snout profile is quite variable, as it can be flat with a rounded tip or short and acuminate. *Telmatobiushalli* presents a smooth skin with minuscule granules, which in some specimens are almost absent on the dorsum. In other cases, they can be more evident on the limbs, flanks, or even covering the ventral surface. These granules become most prominent on the posterior and ventral parts of the thighs. Mature males have very small spines associated with the granules, in addition to conspicuous, black nuptial pads on their thumbs. The coloration of dorsum and extremities can be described as a broad spectrum of brown, olive and yellowish speckles that alternate with dark, almost black spots or marks. Some frogs have fewer dark spots and the brown colors predominate, others show extensive dark areas (Fig. 5A–D). The ventral coloration is lighter, with shades of cream or pink, mixed with yellow areas or white dots (Fig. 5E). A noteworthy character is the light, yellow annulus around the eyes of some specimens (Fig. 6A), a trait that is shared with *T.dankoi* and *T.vilamensis* (JvT, pers. obs.), but seemingly not with other Chilean congeners. Loose skin folds at the posterior part of the thighs can be more or less developed, but seem more frequent in corpulent individuals and mature males. Another highly variable character is the extent of the interdigital membrane. All examined animals had fully webbed toes, but while in some cases the webbing was barely distinguishable towards the tips of the phalanges, others presented very prominent lateral fringes. The tadpoles are large and robust (97.27 mm at Gosner stages 36–37) (Table 3), with a thick, pointed tail (tail length = 1.52× BL; stages 36–37) and show approximately the same pigmentation patterns as adults, but with entirely smooth skin (Fig. 5F).

**Table 2.** Morphometrics of adults of *Telmatobiushalli* from Miño. All measurements are expressed in millimeters. Measurements of the holotype (AMNH A-44753) and one of the paratypes (AMNH A-44754) were taken from Formas et al. (2003); SVL = snout-vent length, HW = head width, HL = head length, IOD = inter-orbital distance, IND = inter-nostril distance, FL = foot length, TL = tibia length.

Adults (n = 11)					
**Variable**	**Mean**	**Min**	**Max**	**Holotype**	**Paratype**
SVL	42.94	38.95	57.15	57.06	48.04
HW	13.34	11.65	19.80	18.75	16.58
HL	12.76	11.00	17.80	16.50	14.27
IOD	4.06	3.30	5.75	6.04	4.91
IND	2.87	2.20	4.20	3.65	3.03
FL	22.15	20.10	29.15	40.21	32.27
TL	18.90	17.00	21.55	24.03	20.26

**Table 3.** Morphometrics of larvae of *Telmatobiushalli* from Miño. All measurements are expressed in millimeters; TTL = total length, BL = body length.

Tadpoles (n = 9)			
**Gosner stage**	**n**	**TTL (Mean)**	**BL (Mean)**
27	1	58.35	24.00
33	1	97.95	30.75
36	5	97.38	38.39
37	2	97.00	39.10

### ﻿DNA sequences and phylogenetic analyses

**Figure 7.**
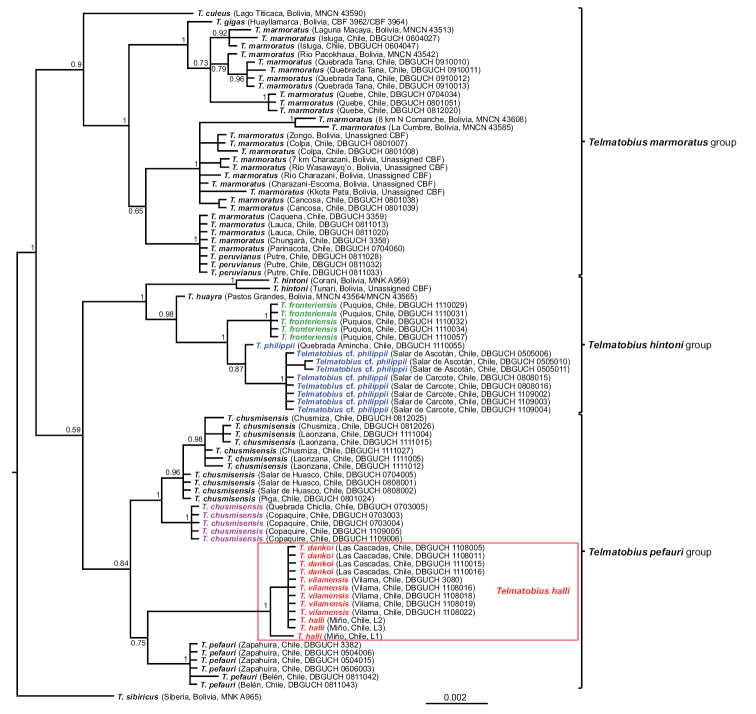
Bayesian consensus tree (50% majority-rule; mitochondrial genes concatenated, treated as two separated partitions), showing the relationships among Chilean *Telmatobius* and the species groups recovered by Sáez et al. (2014). The specimens of the species and populations of the extreme south of the distribution of the genus in Chile are highlighted with the same colors of the map in Fig. 1A. The values next to the nodes correspond to posterior probabilities and the scale bar below the tree represents the expected substitutions per site along the branches. Identification of populations of Copaquire, Quebrada Chiclla, Quebrada Choja, and Aguas Calientes follows the taxonomy prior to Fibla et al. (2018) and Cuevas et al. (2020). The red box indicates the taxonomic changes proposed in this study.

We obtained final alignments of 568 nucleotide sites for the fragment 16S and of 975 for the cytb. However, both alignments were incomplete because the sequences of several specimens included from previous studies are shorter, particularly some fragments of the cytb of the *T.marmoratus* group from De la Riva et al. (2010). The topologies obtained in the Bayesian consensus trees (50% majority-rule) of the analyses with two or four (considering the different codon positions of the cytb) partitions were virtually identical; only slight differences were observed in branch lengths and in a few posterior probability values. The relationships recovered in both analyses were in agreement with those obtained by Sáez et al. (2014) and Fibla et al. (2017), recovering the monophyly of the three species groups (*T.marmoratus*, *T.hintoni*, and *T.pefauri*) present in Chile, although the last one with low support (posterior probability, pp < 0.95) (Fig. 7). Also, the relationships among species and populations within groups are consistent with those studies; for example, the close relationship among populations of Ascotán and Carcote salt flats + *T.philippii* + *T.fronteriensis* and between *Telmatobiuspefauri* Veloso & Trueb, 1976 and the clade made up of *T.dankoi* + *T.vilamensis* (although in this case with low support, pp = 0.75). In our analyses, the three samples of *T.halli* group with *T.dankoi* and *T.vilamensis* with the maximum support (pp = 1). All the specimens of *T.dankoi* (n = 4) and *T.vilamensis* (n = 5) make up a polytomy with two of the tadpoles of *T.halli* (L2 and L3), which constitutes the sister group of the third tadpole (L1) (Fig. 7). The polytomy results from the fact that the sequences of all these specimens are identical in their entirety (the 1,543 sites of both fragments), while the separation of the haplotype of tadpole L1 is due to two differences in the cytb fragment.

### ﻿Synonymy of *T.halli*, *T.dankoi* and *T.vilamensis*

Sáez et al. (2014) were the first to include *T.dankoi* and *T.vilamensis* in a molecular phylogenetic analysis. They obtained identical mitochondrial sequences (genes 16S and cytochrome b) from several specimens of both species and based on their morphological similarity (including some diagnostic characters, see Table 4) suggested that they corresponded to the same species. Fabres et al. (2018) tested 29 microsatellites in several *Telmatobius* species and found the same allele size ranges at various loci in *T.dankoi* and *T.vilamensis*. They note that this is observed only between these two species, supporting the suggestion of Sáez et el. (2014) that both correspond to the same taxon. Here, we obtained mitochondrial sequences from two individuals of *T.halli* that are identical to those of *T.dankoi* and *T.vilamensis* (a third individual differs by only two bases), suggesting a possible synonymy of these three species. The descriptions and diagnoses of *T.halli* (Noble 1938; Formas et al. 2003), *T.dankoi* (Formas et al. 1999) and *T.vilamensis* (Formas et al. 2003) are mainly based on external and osteological features, so considering the identity of the mitochondrial sequences among these three species, it is important to reevaluate the morphological differences that have been described between them. Table 4 compares the traits that have been included in the diagnoses of the three species as they appear in different sources. Below, for each trait, we highlight possible instances of polymorphism as well as the discrepancies that emerge when comparing the different sources and incorporating new observations.

**Table 4.** Phenotypic similarities and differences between *T.halli*, *T.dankoi*, and *T.vilamensis*. Bold font indicates diagnostic characters. Numbers in parentheses specify the source of the information: (1) Noble (1938); (2) Veloso et al. (1982); (3) Formas et al. (1999); (4) Benavides et al. (2002); (5) Formas et al. (2003); (6) Barrionuevo (2017); (7) this study. The traits are described as they appear in the cited sources. In square brackets, some clarifying details that appear in the same source were added. The underlined traits are the differences between *T.dankoi* and *T.vilamensis* described by Formas et al. (2003). Veloso et al. (1982) described the morphology of *T.halli* based on specimens from Calama (*T.dankoi* according to Formas et al. 1999), but also considered the population of Vilama River as that species. Therefore, the characteristics described by those authors should be applicable to all three species. For simplicity, here we include them only in the *T.dankoi* column.

Trait	*T.halli*	*T.dankoi*	*T.vilamensis*
Dentition	vomerine teeth absent (1)	vomerine, **premaxillary, and maxillary teeth absent** (2, 3)	vomerine, **premaxillary and maxillary teeth absent** (5)
	**maxillary teeth rudimentary [0.2–0.3 mm], only present on the upper jaw** (1)	–	teeth present in some individuals (6)
	**premaxillary teeth absent, maxillary teeth rudimentary** (5)	–	–
Skin	smooth (1, 5, 7)	smooth dorsal and ventral skin (2)	smooth; flanks, chest, throat, and ventral surfaces of the arms without keratinous spines (5)
	few granules on dorsum and the posterior surfaces of the thigh, more prominent around and below the vent (1)	**small keratinous spines on head, flanks, posterior third of the dorsum and extremities** [both sexes] (3)	numerous, minute, transparent or white spines on the venter and the ventral surface of the extremities (5)
	flanks, extremities and posterior dorsum with minute granules and dark keratinous spines; the extension of this trait is highly variable (7)	minute dark spines irregularly distributed on flanks, throat and anterior extremities (5)	dark spines on flanks, extremities and posterior dorsum (7)
Postfemoral fold	**absent** (5)	**wide**; well-developed (3)	**present** but smaller (5)
	present; variable in size (7)	–	well-developed in holotype (5)
Snout (dorsal view)	**truncated** (5)	rounded or pointed (5)	rounded or prominently pointed (5)
	varies between truncated and slightly pointed (7)	–	acuminate (4)
Snout (lateral view)	flat (1)	pointed, depressed (2)	strongly **depressed** (5)
	moderately short in lateral view (5)	not depressed (5)	projected distally (4)
	varies between flat and rounded and short and acuminate (7)	–	–
Webbing	toes webbed to the tips but so emarginate that they appear only half webbed (1, 7)	not mentioned, but the illustration of the holotype shows a well-developed webbing that ends in fringes toward the tips (fig. 3F of Formas et al. 1999) (3)	wide fringes (4)
	**toes extensively webbed, outer border of Toe V widely fringed** (5)	–	**toes webbed; outer border of Toe V moderately fringed** (5)
	size of the fringes variable (7)	–	–
Tongue	oval, entire, two-thirds the width of mouth at its greatest transverse diameter (1)	elliptical (2)	**nearly ovoid, elongate, almost adhered to the floor of mouth, posterior border free** (5)
	completely attached to the floor of the mouth (2)	rounded (5)	–
	**round, thick; with posterior border free, unnotched; slightly longer than wide; attached through more than 75% of its length** (5)	–	–
Tympanum	**tympanum absent** (1, 5)	tympanum and tympanic annulus absent (5)	**tympanum and tympanic annulus absen**t (5)
	**tympanic annulus absent** (5)	–	–
Cranial osteology	**choanae large, subrectangular** (5)	cranium well-ossified (5)	**cranium poorly ossified** (5)
	–	**vomer absent** (3)	**vomers rudimentary or absent** (5)
	–	columella absent (3)	**columella absent** (5)
	–	–	**choanae large, circular** (5)
	–	–	**neopalantines reduced** (5)
Tadpoles	long pointed tails; the distal third or two-fifths [of the dorsal fin] is thickly spotted with dark brown (1)	rounded tail end (2)	tail tip rounded (5)
	–	end of tail pointed (3)	myomeres and fins with irregular, dark brown spots (5)
	–	uniformly pigmented tail (2)	–
	–	**distal tip of the tail black** [posterior third] (3)	–
	–	presence of black tip is variable (7)	–
Tibio-tarsal joint carried forward	extends to the posterior margin of the eye (1)	does not reach the posterior border of the eye (3)	reaches or exceeds the posterior border of eye (5)
	reaches the anterior border of the eye (3)	reaches or exceeds the posterior border of eye (5)	–

The rudimentary nature of the maxillary teeth of *T.halli* was one of the features that motivated the description of the species. Since we did not examine the dentition of the frogs from Miño, an evaluation of this issue remains pending. At first glance this point seems decisive, adding the fact that the absence of teeth is also listed as an important trait in the diagnoses of *T.dankoi* and *T.vilamensis*. Nevertheless, Barrionuevo (2017) pointed out that the presence or absence of teeth can vary intraspecifically in some species of *Telmatobius* and even cited *T.vilamensis* as an example where both conditions have been observed.

*Telmatobiusdankoi* was distinguished by having small keratinous spines on the head, flanks, posterior third of the dorsum and extremities in both sexes (Formas et al. 1999), while the skin of *T.halli* and *T.vilamensis* was described as smooth (Noble 1938; Formas et al. 2003). Some of the individuals from Miño had indeed smooth skin, others presented small granules in different densities. We also observed mature males with small black spines mainly on the flanks, extremities, and the posterior part of the dorsum, but in some cases even on the chest and venter. Formas et al. (2003) differentiate *T.vilamensis* from *T.dankoi* alluding to the skin being smooth in the former and spiny in the latter, yet they also state that the holotype of *T.vilamensis* has numerous, minute, transparent or white spines on the venter and the ventral surface of the extremities. We show an individual of *T.vilamensis* that presents small black spines on flanks, extremities, and posterior dorsum (Figure 6C; Felipe Rabanal, pers. comm.). It is important to note that Veloso et al. (1982) indicate that the adults of *T.halli* from the Loa River at Calama (later described as *T.dankoi* by Formas et al. 1999, type locality Las Cascadas) have “smooth dorsal and ventral skin”, in clear contrast to what appears in the description of *T.dankoi*. Furthermore, they do not mention any difference in skin texture between the population of Calama and that of Vilama River (*T.vilamensis*), which they also consider *T.halli*. In any case, interpopulational variation of the skin texture is not a novelty in the genus, as it has been reported for *T.rubigo* (Barrionuevo and Baldo 2009).

Another feature on which emphasis was made in the descriptions of *T.dankoi* and *T.vilamensis* is the presence of postfemoral folds. Both species differ from their congeners by presenting well-developed folds, although it was reported that these are smaller and thinner in *T.vilamensis* (Formas et al. 2003). For *T.halli*, Noble (1938) does not mention anything about this trait in the original description, but Formas et al. (2003), in the redescription of the species, explicitly indicate the lack of these folds. However, in the photographs of the holotype (Fibla et al. 2018: fig. 6A; Cuevas et al. 2020: fig. 3E, F) this trait seems to be present. The paratype depicted in Fibla et al. (2018: fig. 6B) does not have folds, which suggests that this would also be an intraspecific polymorphism. All of the adults that we observed in Miño, both males and females, presented postfemoral folds, although they seem more developed in males.

In the case of *T.vilamensis*, the shape of the snout was stated as an outstanding character and is described as being “strongly depressed” (Formas et al. 2003). Noble (1938) used the term “flat” for the snout of the holotype of *T.halli*, while Formas et al. (2003) described the snout of the same specimen as truncated in dorsal and short in lateral view. On the other hand, Veloso et al. (1982) mentioned a pointed and depressed snout for the frogs from Calama (as *T.halli*), while Formas et al. (2003) clearly categorized the snouts of the animals from the same population as “not depressed” (as *T.dankoi*). Veloso et al. (1982) do not mention differences in the shape of the snout (pointed, depressed) between the populations of Calama and Vilama River, which is contradicted by Formas et al. (2003), who include the shape of the snout among the few traits that distinguish *T.dankoi* of *T.vilamensis* (not depressed versus strongly depressed, respectively). In Miño, we observed variable snout lengths and forms, and some degree of variation in this feature is to be expected as well in the Vilama River and Las Cascadas.

Almost half of the diagnostic characters of *T.vilamensis* are cranial bone structures, which contrasts with the diagnoses of *T.halli* and *T.dankoi*, where few osteological characters were included. Therefore, from an osteological point of view, there are not many elements to compare the three populations. Some of the aforementioned osteological features have been attributed to immature stages of post-metamorphic development in *T.dankoi* and *T.vilamensis*, in comparison to other species of the genus (Barrionuevo 2013). Barrionuevo (2017) presents two possible explanations for this interspecific variation: i) Formas et al. (2003) used immature individuals for their osteological analysis, or ii) the differences in the analyzed attributes are produced by phenotypic plasticity. Since the specimens used for the descriptions of the skeletons of *T.dankoi* (Formas et al. 1999) and *T.vilamensis* (Formas et al. 2003) were explicitly stated to be adults, the second explanation would be more likely. However, neither of these cases can assure a reliable species delimitation. Regarding the different degrees of cranial ossification in *T.dankoi* and *T.vilamensis* (Formas et al. 2003), it is necessary to consider that it might correspond to intraspecific variation, as described for *T.oxycephalus* (Barrionuevo 2013).

The development of webbing and fringes on the toes has been included in the diagnoses of *T.halli* and *T.vilamensis* (Formas et al. 2003), but the differences described for these traits are only of degree, are very subtle, or vary within the population of Miño. Another feature that appears in diagnoses is the shape of the tongue. In fact, it is one of the characteristics with which Formas et al. (2003) differentiate *T.vilamensis* from *T.dankoi*. However, the description of the form of the tongue of *T.dankoi* varies between sources, being the description of Veloso et al. (1982) very similar to that of *T.vilamensis* (Table 4). The absence of the tympanum and tympanic annulus has also been included in the diagnoses of *T.halli* and *T.vilamensis*, but in this case all three species lack these structures.

With respect to the tadpoles, Formas et al. (1999) mentioned that those of *T.dankoi* have the tip of the tail black; however, when Veloso et al. (1982) described the appearance of larvae from the same population, they mentioned a “uniformly pigmented tail” and did not say anything about such a conspicuous mark (Table 4). We also endorse that at least some tadpoles from Las Cascadas do not exhibit dark tail tips (JvT, pers. obs.). Between the two sources listed above, there is also a discrepancy for the same species in the description of the shape of the tip of the tail.

One particular character was not included in the diagnosis of any of the three species, but was used to differentiate *T.dankoi* from its Chilean congeners (Formas et al. 1999) and in the dichotomous key to adults of the *Telmatobius* species from Chile (Formas et al. 2003). It refers to the condition of the tibio-tarsal joint not reaching the eye when bent forward in *T.dankoi*. Interestingly, in the key, the same species was categorized as having a tibio-tarsal joint that “reaches or exceeds the posterior border of [the] eye” (Table 4). This could mean that this attribute is polymorphic or, otherwise, that the authors might have overlooked this detail.

The usefulness of three additional characters that appear in the diagnoses can be discarded. Body size was included in the diagnosis of *T.dankoi* (SVL = 49.7–51.7 mm, Formas et al. 1999), but this range is within the size limits of the *T.halli* type series (42–57 mm, Noble 1938) and overlaps with the size range of *T.vilamensis* (38.36–50.81 mm, Formas et al. 2003). All of these ranges of values fall within the size limits measured by us in the Miño individuals (Table 2). Another feature included in the diagnosis of *T.vilamensis* is the number and shape of the chromosomes: 26 bi-armed chromosomes (Formas et al. 2003). However, these same authors point out that the karyotype of *Telmatobius* species is uniform, with 26 chromosomes and a fundamental number of 52, and that in all described cases the secondary constriction always is found in the short arm of pair 6. Finally, the coloration of the dorsum was included in the diagnoses of *T.vilamensis* and *T.halli* (Formas et al. 2003). In general, the coloration of the frogs from the three focal localities (Miño, Las Cascadas, and Vilama River) is very similar (Fig. 6A–C). The size and number of darker spots vary between individuals of the same population, but this variation is subtle if compared to the differences that can be found in other *Telmatobius* species. Just to give examples of this in species and populations from Chile, intraspecific heterogeneity in body coloration is described for *T.marmoratus* (Veloso et al. 1982) and well-illustrated for *T.marmoratus* (Sáez et al. 2014: fig. 3), *T.pefauri* (Fibla et al. 2017: fig. 4 A–E) and *T.chusmisensis* (Fibla et al. 2018: fig. 5A–C).

In summary, considering all the available information, in the literature there are two contrasting views on morphological variation among *T.halli*, *T.dankoi*, and *T.vilamensis*. On the one hand, there are two studies where the limits of *T.halli* are broadened: one that adds the population of Arroyo Vilama (Cei 1962) and another that includes this same population and that of Calama (Veloso et al. 1982). In both studies, no morphological differences between these populations were described. On the other hand, there are two studies that focus on dissimilarities observed in frogs from these localities and split the populations into separate species (Formas et al. 1999, 2003). Combining the information from the different sources, including novel observations made here, differences and discrepancies arise in the descriptions of many traits for the same species (Table 4). In the cases where the information is specified, authors examined different collection specimens from the same populations (Veloso et al. 1982; Formas et al. 1999, 2003), so the differences observed among them can be interpreted as polymorphisms, which in many cases are shared with the other populations. This applies to most of the characters that were described as diagnostic for one or more species: skin texture (presence/absence of spines), snout shape, webbing and fringes of the toes, tongue shape, shape, and pigmentation of the end of the tadpole’s tail, and extension of the tibio-tarsal joint when extends forward. The presence of postfemoral folds and the absence of the tympanum and tympanic annulus, apply to all three species, while the presence/absence of teeth is a polymorphism in *T.vilamensis* (Barrionuevo 2017) that could be shared with the other species. This leaves as diagnostic differences only the degree of ossification of the skull (between *T.dankoi* and *T.vilamensis*) and a slight distinction in the shape of the choanae, but these osteological observations are based on a limited number of specimens (two of *T.dankoi* and three of *T.vilamensis* in the case of the skull). Furthermore, as mentioned above, variation in osteological characters has been described within some *Telmatobius* species, including the degree of ossification (Barrionuevo 2013 and references therein). Regardless of the position adopted (morphological uniformity among populations or widespread polymorphism), neither of the two supports the distinction of species.

Therefore, the external, osteological, and ecological characteristics as a whole do not allow to clearly distinguish *T.halli*, *T.dankoi*, and *T.vilamensis*, and the described variation of some morphological characters can be interpreted as intra- and interpopulation polymorphisms between the three known populations. Bearing in mind also their indistinguishable mitochondrial sequences and their high genetic affinity detected with microsatellite markers (Fabres et al. 2018), we herein propose to consider *T.dankoi* and *T.vilamensis*, by nomenclatural precedence, junior synonyms of *T.halli*. We further suggest adopting the vernacular name of *T.dankoi* (Loa Water Frog), as it has gained popularity (Lobos and Rojas 2020) and would represent the species appropriately.

## ﻿Discussion

The discovery of a *Telmatobius* population at the origin of the Loa River (Miño) definitely solves one of the most persistent enigmas of Chilean herpetology: the location of the population originally described as *T.halli*. This riddle persisted for more than eighty years because of the uncritical acceptance of the inherently vague description of the type locality by Noble (1938). However, the solution came from a careful analysis of publications and other documentary sources where some of the members of the IHAEC described their activities and the place and circumstances in which the amphibians were collected (Correa 2021). It can be argued that both Fibla et al. (2018) and Cuevas et al. (2020) applied that same strategy, but paradoxically both reached different (and incorrect) conclusions about the location and identity of the species. In the case of Fibla et al. (2018), some key sources in which the place is explicitly described were not consulted, while in the case of Cuevas et al. (2020), more importance was given to the characteristics of the place they hypothesized as the type locality (Aguas Calientes) than to the information contained in the documentary sources.

Cuevas et al. (2020) defined five conditions to validate Aguas Calientes as the type locality of *T.halli*: “1) the place should be a thermal spring, 2) presence or ruins of a concrete swimming pool in the area, 3) have a small oasis with abundant vegetation, 4) be 3,000 ft (~ 900 m) down of Collahuasi (Montt) and 5) be located near to Ollagüe.” The measurements and observations made here show that Miño fulfills the first four conditions, while the new sources consulted confirm that the fifth one does not apply to *T.halli*.

The Loa River at Miño has an almost constant water temperature of around 20 °C, even at prolonged ambient temperatures below the freezing point, which suggests geothermal activity and matches well with the literature on the type locality of *T.halli* (Keys 1936a, 1936b; Noble 1938; Dill 1980). This temperature is similar to that of the collection site in Copaquire (19 °C), the purported type locality of *T.halli* according to Fibla et al. (2018), but lower than that of Aguas Calientes (27.7 °C). Only two other species of *Telmatobius* are known to inhabit warm or thermal waters, *T.fronteriensis* in Puquios (originally found in a small thermal pond with a water temperature of 22.9 °C, Benavides et al. 2002) and T.cf.philippii in several springs at the Ascotán Salt Flat (Lobos et al. 2018). In the latter case, the water temperature varies widely between springs and seasons, but in general, spring water has medium to high temperatures compared to the air. In contrast to the water temperature, at Miño we observed a significant fluctuation range between the air temperatures at day and those at night (34.9 °C), which could have been even higher, considering that we did not log the temperature data for the entire day. The constant water temperature may benefit the species, as it serves as a buffer for the thermal oscillations of the environment and prevents thermal stress. Nevertheless, the temperature might not be constant throughout the year, as snowmelt increases the flow rate during thaw season.

We were able to verify on the ground two other conditions defined by Cuevas et al. (2020): the presence of ruins of a concrete swimming pool and an oasis of vegetation. Although Cuevas et al. (2020) point out that there is a pool in Aguas Calientes that was built in 2012 on a previous construction, we show that in Miño there are the ruins corresponding to the concrete pool where the members of the IHAEC swam on 23 June 1935 (Fig. 3). Regarding the oasis of vegetation, we only have the description of Dill (1980) (“a flourishing green oasis”) and that of McFarland’s diary (“green valley”). Both descriptions fit well the current appearance of the area where the Loa River is born (Figs 1C, 2B), suggesting that the vegetation of the place has not changed much since the time of the expedition. The vegetation extends along the riverbed for more than a kilometer upstream from the ruins of the swimming pool and continues downstream along the Loa river canyon, so the place is much more than only a “small oasis” as described by Cuevas et al. (2020).

The elevation of the type locality of *T.halli* is one of the problematic aspects of the description provided by Noble (1938). Fibla et al. (2018) estimated that the site would be at ~ 4,000 m a.s.l., considering only the indications given by Dill (1979), while Cuevas et al. (2020) indicated that Aguas Calientes is located at 3,717 m. The altitude of our study site at Miño (3,900 m) was not measured in situ but obtained from Google Earth. Neither of these values matches the one Noble (1938) pointed out in the species description: 10,000 ft (3,048 m). Cuevas et al. (2020) tried to explain this difference by alluding to an underestimation of 610 m in the actual elevation of the Aucanquilcha mine that was reported by members of the expedition (e.g., Keys 1936b; Keys et al. 1938). However, that explanation does not take into account that the data for most of the other reported places (Chuquicamata, Ollagüe, Collahuasi, and Punta de Cerro) differ by less than 100 m from the altitudes that can be obtained, for example, from Google Earth. To further complicate this matter, the four chronicles that describe the Sunday trip to the source of the Loa River indicate different heights for that place. In fact, David B. Dill provided two different heights for Montt, 16,400 ft (~ 4,999 m) and 15,440 ft (~ 4,706 m) (Dill 1979, 1980, respectively), although in both cases he indicated that the site where the concrete pool was located was 3,000 ft (~ 916 m) lower. Ancel Keys instead specified the heights of the pool directly: 3,700 m (Keys 1936a) and 12,000 ft (~ 3,658 m; Keys 1936b). All these measurements should not be taken as absolute, as they seem a little roughly estimated and were indeed stated in more anecdotal parts of the publications. Among all the available values, the statement of Dill (1980) (12,440 ft = ~ 3,792 m) is the closest to that of Miño and it is further supported as his specification of the height of the former camp of the Collahuasi mine at the Montt railroad station (20°58'35"S, 68°41'20"W) matches very well the value from Google Earth. This explanation still does not solve the reason why Noble (1938) established that the type locality was at 10,000 ft above sea level, yet it only adds to the general impreciseness of the geographic information in his description.

The fifth condition of Cuevas et al. (2020), the proximity to Ollagüe, is the other problematic aspect of the description of the type locality given by Noble (1938) because there is no place that could be considered close to this town that is at 10,000 ft. In fact, the closest places to Ollagüe with that altitude are ~ 70 km to the west (in a straight line), on the other side of the Loa River. This is one of the reasons why searches for the species around Ollagüe were unsuccessful (Formas et al. 2003, 2005; IUCN 2015) and why Cuevas et al. (2020) concluded that the type locality is located only 12 km southwest of that town (Fig. 1A), but at a higher altitude. Thus, the suggestion from Fibla et al. (2018) that Noble might have used the location from which the specimens were sent as the type locality seems plausible to us.

In the chronicles of the IHAEC, there is little information about the population of *Telmatobius* from Miño. Dill (1979, 1980) only mentions that Frank G. Hall collected some specimens and that one of them proved to be a new species of amphibian. Keys (1936a) gives more details, indicating that many frogs and tadpoles were found in temperate ponds. This is consistent with the description of the species since the type series consists of five adult females, one immature female and six tadpoles. Currently, there seems to exist an abundant and healthy population, as frogs were found at several spots along the stream. This suggests that the environmental conditions at the site are similar to those at the time of the expedition. Regarding the individuals observed now in Miño, adults and tadpoles present external characteristics congruent with the description of *T.halli* (Noble 1938). Adults have almost completely smooth skin and a long and flattened snout. In addition, the general coloration pattern (brown and olive with darker irregular spots) and the size of the adults and tadpoles is compatible with the type series.

This is the first study to include the population that was originally described as *T.halli* in a phylogenetic analysis. Although this analysis was performed only with mitochondrial sequences (nuclear markers have not yet been included in phylogenetic analyses of the genus), it included all Chilean species of the genus and all known populations that are geographically close. *Telmatobiushalli* formed a highly supported clade with the two endemic species from the extreme south of the genus distribution in Chile, *T.dankoi* and *T.vilamensis*, both of which had previously been considered *T.halli* (e.g., Cei 1962; Veloso et al. 1982). Furthermore, within the clade there was no resolution among those species since the sequences (both genes) of two of the three specimens of *T.halli* (L2 and L3) are identical to those of the other two species. The third specimen (L1) shows two differences in cytochrome b with respect to all the specimens that make up the clade. This result, together with an exhaustive and detailed analysis of the morphological information (see last section of Results), lead us to the conclusion that *T.halli*, *T.dankoi*, and *T.vilamensis* are conspecific.

The possible synonymy between *T.dankoi* and *T.vilamensis* had already been pointed out by Sáez et al. (2014) and was reaffirmed by Fabres et al. (2018) based on genetic data (microsatellites). The populations previously assigned to those species not only share identical mitochondrial sequences with *T.halli*, but they also have common morphological characteristics that support their close affinity (e.g., coloration, size, flattened snout, presence of postfemoral folds, absence of vomers). This high morphological similarity explains why the populations of “Ollagüe” (actually Miño), Arroyo Vilama (Vilama River) and Calama (Las Cascadas) were previously reunited under the name *T.halli* (Cei 1962; Veloso et al. 1982) and our reevaluation of all morphological information shows that the majority of the diagnostic characters that supported the differences between *T.halli*, *T.dankoi*, and *T.vilamensis* would correspond to polymorphic traits. The geographic context is also relevant in this case. *Telmatobiushalli* inhabits the same watershed (Loa River) as the population previously assigned to *T.dankoi*, while the only known population of the former *T.vilamensis* is found in the Salar de Atacama basin, which adjoins the Loa River basin in the southeast (Fig. 1A).

The conspecificity of these three species also has important consequences for the conservation of these populations due to the current critical situation of the populations previously assigned to *T.dankoi* and *T.vilamensis*. In 2019, the only stream from where *T.dankoi* was known (Las Cascadas) almost completely dried up, resulting in the loss of approximately 90% of the total population (Lobos and Rojas 2020). In addition to that, individuals assigned to *T.vilamensis* have not been observed again in the Vilama River (the only locality attributed to that species) after a flash flood destroyed the site in 2016 (Lobos and Rojas 2020). Therefore, Miño is the only known locality for this taxon with an apparently large population and an unaltered environment. In 2005, the Chilean government started the legal process for the creation of the National Reserve Alto Loa, but the efforts were not carried on and this protected area does not exist yet (Tomás Gerö, CONAF, pers. comm.). Our findings could be an excellent opportunity to resume the task, especially taking into consideration the attention which *T.dankoi* received in the international media in 2019 (Lobos and Rojas 2020).

Currently, based on the scarce and incomplete information available for this species, *T.halli* is listed as Data Deficient by the IUCN (IUCN SSC Amphibian Specialist Group 2015) and as Critically Endangered by the Chilean government (MMA 2019). For this last categorization, in which the uncertainty of the location of the type locality is recognized, it was assumed that the species: has an area of occupancy of ~ 1 km^2^, is known from a single locality and its habitat is deteriorating due to excessive use of water and the threat of the chytrid fungus (according to observations in other species of the genus in Chile). In this context, the description of the new population of Miño and the environment where it inhabits, as well as the proposed taxonomic change, constitute fundamental information to reassess the conservation status of *T.halli*, but several aspects must be investigated in greater depth.

Up until now, there seems to exist very little anthropic disturbance at the location described in this work and the place appears to be visited only occasionally by anglers, off-road enthusiasts, and mountaineers. Since the times of the IHAEC, it has been a recreational area mainly for the mine staff and, according to locals, still in the 1990s, the spot was sporadically visited by workers from the nearby mines. Accessing the site is very difficult and an increase in tourist activity is unlikely to happen. The valley is very pristine with no visible pollution signs. There is a vehicular track, that crosses the riverbed, which means that there could occur an occasional roadkill or minor contamination with motor oil or fuel; however, given the remoteness of the location not many vehicles pass through. Furthermore, it has yet to be evaluated if the intense nearby mining activity at the Collahuasi and Quebrada Blanca copper mines poses an imminent threat to the *Telmatobius* population, for instance, if contamination with heavy metals could occur through industrial dust dispersion (e.g., Csavina et al. 2012) or if potential upcoming projects demand water extraction for mining processes.

As mentioned above, other species of *Telmatobius* have been found infected with *Batrachochytriumdendrobatidis* (Bd) in northernmost Chile and there is an ongoing spread of chytridiomycosis southwards the Andes (Solís et al. 2015). Now that the type locality of *T.halli* has been rediscovered, most certainly other herpetologists will visit the place and special attention has to be taken to avoid contamination with the pathogen. Precaution is even more imperative, given the fact that Miño is at the headwaters of the Loa River and Bd could easily expand to other putative populations downstream (Johnson and Speare 2005).

Introduced salmonids are another threat for native amphibians in Chile (Soto-Azat et al. 2015). A recent study (Lobos et al. 2020) reports the presence of *Oncorhynchusmykiss* (Walbaum, 1792) in several locations of the upper Loa, being Sapunta the nearest sampling point to the source (approx. 18 km). No salmonids were detected during the fieldwork, but given that they already have colonized the rest of the river, their presence in Miño is quite probable. The highest record of the rainbow trout in the mentioned study is 4,560 m in Misitune (18°22'S), which means that elevation would not be an impediment to the potential expansion of the invasive fish into the habitat of *T.halli*.

Besides these anthropic influences, it is also necessary to consider natural factors that could constitute a threat to the population. The extreme north of Chile is affected by intense precipitations during the so-called Altiplanic winter, which generates flash floods and landslides, having a negative impact on the biota. This phenomenon significantly reduces the riparian vegetation (Paicho-Hidalgo et al. 2015) on which the frogs of the genus *Telmatobius* heavily depend, probably for shelter from UV radiation and predators. An example of this type of catastrophic event is what happened in the Vilama River (see above). A similar case is Quebrada de Amincha, type locality of *T.philippii*, where the effects of a recent swelling of the creek were observed in February 2018 (JvT, pers. obs.). The vegetation was severely damaged, and even though a few living specimens were detected, it took a long time to locate them. Paicho-Hidalgo et al. (2015) pointed out that the ecological resilience of these ecosystems allows quick revegetation, but still, such an event in the habitat of *T.halli* could diminish the population and make it more vulnerable to other stresses. Comparing the current rock formations to those from the 1935 recordings suggests that destructive erosion events do occur in Miño.

All these threats, alone or in combination, could potentially lead to the extinction of *T.halli*. Therein lies the importance of protecting the upper portion of the Loa River, where the watercourse and the surroundings are seemingly untouched for several kilometers. As a next step, surveys to determine the presence of *Telmatobius* along the river and its tributaries should be organized.

## ﻿Appendix 1

Specimens of *Telmatobius* included in the phylogenetic analyses. For each specimen, species, locality (country), collection number or label, GenBank accession number (for each DNA fragment) and source of the sequences are indicated. Identification of specimens follows the taxonomy prior to Fibla et al. (2018), Cuevas et al. (2020) and this study (in the case of *T.dankoi* and *T.vilamensis*). The names of the localities are in Spanish as they appear in the respective publications.

**Table:**

Species	Locality	Collection number or label	cytb	16S	Source
*T.culeus*	Lago Titicaca (Bolivia)	MNCN 43590	GU060589	GU060554	De la Riva et al. (2010)
*T.gigas*	Huayllamarca (Bolivia)	CBF 3962 (cytb) / CBF 3964 (16S)	GU060593	GU060558	De la Riva et al. (2010)
*T.marmoratus*	Laguna Macaya (Bolivia)	MNCN 43513	GU060600	GU060565	De la Riva et al. (2010)
*T.marmoratus*	Isluga (Chile)	DBGUCH 0604027	KJ562938	KJ563008	Sáez et al. (2014)
*T.marmoratus*	Isluga (Chile)	DBGUCH 0604047	KJ562939	KJ563009	Sáez et al. (2014)
*T.marmoratus*	Río Pacokhaua (Bolivia)	MNCN 43542	GU060602	GU060567	De la Riva et al. (2010)
*T.marmoratus*	Quebrada Tana (Chile)	DBGUCH 0910010	KJ562944	KJ563014	Sáez et al. (2014)
*T.marmoratus*	Quebrada Tana (Chile)	DBGUCH 0910011	KJ562945	KJ563015	Sáez et al. (2014)
*T.marmoratus*	Quebrada Tana (Chile)	DBGUCH 0910012	KJ562946	KJ563016	Sáez et al. (2014)
*T.marmoratus*	Quebrada Tana (Chile)	DBGUCH 0910013	KJ562947	KJ563017	Sáez et al. (2014)
*T.marmoratus*	Quebe (Chile)	DBGUCH 0704034	KJ562941	KJ563011	Sáez et al. (2014)
*T.marmoratus*	Quebe (Chile)	DBGUCH 0801051	KJ562942	KJ563012	Sáez et al. (2014)
*T.marmoratus*	Quebe (Chile)	DBGUCH 0812020	KJ562943	KJ563013	Sáez et al. (2014)
*T.marmoratus*	8 km N Comanche (Bolivia)	MNCN 43608	GU060603	GU060568	De la Riva et al. (2010)
*T.marmoratus*	La Cumbre (Bolivia)	MNCN 43585	GU060605	GU060570	De la Riva et al. (2010)
*T.marmoratus*	Zongo (Bolivia)	Unassigned CBF	GU060607	GU060572	De la Riva et al. (2010)
*T.marmoratus*	Colpa (Chile)	DBGUCH 0801007	KJ562896	KJ562971	Sáez et al. (2014)
*T.marmoratus*	Colpa (Chile)	DBGUCH 0801008	KJ562897	KJ562972	Sáez et al. (2014)
*T.marmoratus*	7 km Charazani (Bolivia)	Unassigned CBF	GU060608	GU060573	De la Riva et al. (2010)
*T.marmoratus*	Río Wasawayq’o (Bolivia)	Unassigned CBF	GU060610	GU060575	De la Riva et al. (2010)
*T.marmoratus*	Río Charazani (Bolivia)	Unassigned CBF	GU060609	GU060574	De la Riva et al. (2010)
*T.marmoratus*	Charazani-Escoma (Bolivia)	Unassigned CBF	GU060611	GU060576	De la Riva et al. (2010)
*T.marmoratus*	Kkota Pata (Bolivia)	Unassigned CBF	GU060612	GU060577	De la Riva et al. (2010)
*T.marmoratus*	Cancosa (Chile)	DBGUCH 0801038	KJ562889	KJ562964	Sáez et al. (2014)
*T.marmoratus*	Cancosa (Chile)	DBGUCH 0801039	KJ562890	KJ562965	Sáez et al. (2014)
*T.marmoratus*	Caquena (Chile)	DBGUCH 3359	KJ562891	KJ562966	Sáez et al. (2014)
*T.marmoratus*	Lauca (Chile)	DBGUCH 0811013	KJ562892	KJ562967	Sáez et al. (2014)
*T.marmoratus*	Lauca (Chile)	DBGUCH 0811020	KJ562893	KJ562968	Sáez et al. (2014)
*T.marmoratus*	Chungará (Chile)	DBGUCH 3358	KJ562894	KJ562969	Sáez et al. (2014)
*T.marmoratus*	Parinacota (Chile)	DBGUCH 0704060	KJ562895	KJ562970	Sáez et al. (2014)
*T.marmoratus*	Putre (Chile)	DBGUCH 0811028	KJ562898	KJ562973	Sáez et al. (2014)
*T.marmoratus*	Putre (Chile)	DBGUCH 0811032	KJ562899	KJ562974	Sáez et al. (2014)
*T.marmoratus*	Putre (Chile)	DBGUCH 0811033	KJ562900	KJ562975	Sáez et al. (2014)
*T.hintoni*	Corani (Bolivia)	MNK A959	GU060594	GU060558	De la Riva et al. (2010)
*T.hintoni*	Tunari (Bolivia)	Unassigned CBF	GU060596	GU060561	De la Riva et al. (2010)
*T.huayra*	Pastos Grandes (Bolivia)	MNCN 43564 (cytb) / MNCN 43565 (16S)	GU060599	GU060563	De la Riva et al. (2010)
*T.fronteriensis*	Puquios (Chile)	DBGUCH 1110029	KJ562884	KJ562959	Sáez et al. (2014)
*T.fronteriensis*	Puquios (Chile)	DBGUCH 1110031	KJ562885	KJ562960	Sáez et al. (2014)
*T.fronteriensis*	Puquios (Chile)	DBGUCH 1110032	KJ562886	KJ562961	Sáez et al. (2014)
*T.fronteriensis*	Puquios (Chile)	DBGUCH 1110034	KJ562887	KJ562962	Sáez et al. (2014)
*T.fronteriensis*	Puquios (Chile)	DBGUCH 1110057	KJ562888	KJ562963	Sáez et al. (2014)
*T.philippii*	Quebrada Amincha (Chile)	DBGUCH 1110055	KJ562901	KJ562976	Sáez et al. (2014)
Telmatobiuscf.philippii	Salar de Ascotán (Chile)	DBGUCH 0505006	KJ562912	KJ562986	Sáez et al. (2014)
Telmatobiuscf.philippii	Salar de Ascotán (Chile)	DBGUCH 0505010	KJ562913	KJ562987	Sáez et al. (2014)
Telmatobiuscf.philippii	Salar de Ascotán (Chile)	DBGUCH 0505011	KJ562914	KJ562988	Sáez et al. (2014)
Telmatobiuscf.philippii	Salar de Carcote (Chile)	DBGUCH 0808015	KJ562925	KJ562995	Sáez et al. (2014)
Telmatobiuscf.philippii	Salar de Carcote (Chile)	DBGUCH 0808016	KJ562926	KJ562996	Sáez et al. (2014)
Telmatobiuscf.philippii	Salar de Carcote (Chile)	DBGUCH 1109002	KJ562927	KJ562997	Sáez et al. (2014)
Telmatobiuscf.philippii	Salar de Carcote (Chile)	DBGUCH 1109003	KJ562928	KJ562998	Sáez et al. (2014)
Telmatobiuscf.philippii	Salar de Carcote (Chile)	DBGUCH 1109004	KJ562929	KJ562999	Sáez et al. (2014)
*T.chusmisensis*	Chusmiza (Chile)	DBGUCH 0812025	KJ562873	KJ562952	Sáez et al. (2014)
*T.chusmisensis*	Chusmiza (Chile)	DBGUCH 0812026	KJ562874	KJ562953	Sáez et al. (2014)
*T.chusmisensis*	Laonzana (Chile)	DBGUCH 1111004	KJ562919	KJ562989	Sáez et al. (2014)
*T.chusmisensis*	Laonzana (Chile)	DBGUCH 1111015	KJ562922	KJ562992	Sáez et al. (2014)
*T.chusmisensis*	Chusmiza (Chile)	DBGUCH 1111027	KJ562875	KJ562954	Sáez et al. (2014)
*T.chusmisensis*	Laonzana (Chile)	DBGUCH 1111005	KJ562920	KJ562990	Sáez et al. (2014)
*T.chusmisensis*	Laonzana (Chile)	DBGUCH 1111012	KJ562921	KJ562991	Sáez et al. (2014)
*T.chusmisensis*	Salar de Huasco (Chile)	DBGUCH 0704005	KJ562935	KJ563005	Sáez et al. (2014)
*T.chusmisensis*	Salar de Huasco (Chile)	DBGUCH 0808001	KJ562936	KJ563006	Sáez et al. (2014)
*T.chusmisensis*	Salar de Huasco (Chile)	DBGUCH 0808002	KJ562937	KJ563007	Sáez et al. (2014)
*T.chusmisensis*	Piga (Chile)	DBGUCH 0801024	KJ562940	KJ563010	Sáez et al. (2014)
*T.chusmisensis*	Quebrada Chiclla (Chile)	DBGUCH 0703005	KJ562930	KJ563000	Sáez et al. (2014)
*T.chusmisensis*	Copaquire (Chile)	DBGUCH 0703003	KJ562931	KJ563001	Sáez et al. (2014)
*T.chusmisensis*	Copaquire (Chile)	DBGUCH 0703004	KJ562932	KJ563002	Sáez et al. (2014)
*T.chusmisensis*	Copaquire (Chile)	DBGUCH 1109005	KJ562933	KJ563003	Sáez et al. (2014)
*T.chusmisensis*	Copaquire (Chile)	DBGUCH 1109006	KJ562934	KJ563004	Sáez et al. (2014)
*T.dankoi*	Las Cascadas (Chile)	DBGUCH 1108005	KJ562880	KJ562955	Sáez et al. (2014)
*T.dankoi*	Las Cascadas (Chile)	DBGUCH 1108011	KJ562881	KJ562956	Sáez et al. (2014)
*T.dankoi*	Las Cascadas (Chile)	DBGUCH 1110015	KJ562882	KJ562957	Sáez et al. (2014)
*T.dankoi*	Las Cascadas (Chile)	DBGUCH 1110016	KJ562883	KJ562958	Sáez et al. (2014)
*T.vilamensis*	Río Vilama (Chile)	DBGUCH 3080	KJ562902	KJ562977	Sáez et al. (2014)
*T.vilamensis*	Río Vilama (Chile)	DBGUCH 1108016	KJ562903	KJ562978	Sáez et al. (2014)
*T.vilamensis*	Río Vilama (Chile)	DBGUCH 1108018	KJ562904	KJ562979	Sáez et al. (2014)
*T.vilamensis*	Río Vilama (Chile)	DBGUCH 1108019	KJ562905	KJ562980	Sáez et al. (2014)
*T.vilamensis*	Río Vilama (Chile)	DBGUCH 1108022	KJ562906	KJ562981	Sáez et al. (2014)
*T.halli*	Miño (Chile)	L1	OL412556	OL412559	This study
*T.halli*	Miño (Chile)	L2	OL412557	OL412560	This study
*T.halli*	Miño (Chile)	L3	OL412558	OL412561	This study
*T.pefauri*	Zapahuira (Chile)	DBGUCH 3382	KJ562908	KJ562982	Sáez et al. (2014)
*T.pefauri*	Zapahuira (Chile)	DBGUCH 0504006	KJ562909	KJ562983	Sáez et al. (2014)
*T.pefauri*	Zapahuira (Chile)	DBGUCH 0504015	KJ562910	KJ562984	Sáez et al. (2014)
*T.pefauri*	Zapahuira (Chile)	DBGUCH 0606003	KJ562911	KJ562985	Sáez et al. (2014)
*T.pefauri*	Belén (Chile)	DBGUCH 0811042	KJ562923	KJ562993	Sáez et al. (2014)
*T.pefauri*	Belén (Chile)	DBGUCH 0811043	KJ562924	KJ562994	Sáez et al. (2014)
*T.sibiricus*	Siberia (Bolivia)	MNK A965	GU060615	GU060580	De la Riva et al. (2010)

## Supplementary materials

Supplementary material 1 Video S1. Warm springs swimming bath at source of Río Loa in Chile, 1935. Data type Video file Explanation note Footage taken by Ross McFarland during the International High Altitude Expedition to Chile in 1935. The film shows the expedition members on a Sunday trip to the source of the Loa River. On this occasion, Frank Gregory Hall collected the type series of *Telmatobiushalli*. The video is courtesy of Wright State University Libraries' Special Collections & Archives. https://binary.pensoft.net/file/626354This dataset is made available under the Open Database License (http://opendatacommons.org/licenses/odbl/1.0/). The Open Database License (ODbL) is a license agreement intended to allow users to freely share, modify, and use this Dataset while maintaining this same freedom for others, provided that the original source and author(s) are credited.Ross McFarland

## References

[B1] Altig R (2007) A primer for the morphology of anuran tadpoles. Herpetological Conservation and Biology 2(1): 71–74. https://www.herpconbio.org/Volume_2/Issue_1/Altig_2007b.pdf

[B2] Barrionuevo JS (2013) Osteology and Postmetamorphic Development of *Telmatobiusoxycephalus* (Anura: Telmatobiidae) with an Analysis of Skeletal Variation in the Genus. Journal of Morphology 274: 73–96. 10.1002/jmor.20079 23023874

[B3] Barrionuevo JS (2017) Frogs at the summits: phylogeny of the Andean frogs of the genus *Telmatobius* (Anura,Telmatobiidae) based on phenotypic characters. Cladistics 33: 41–68. 10.1111/cla.12158 34710971

[B4] Barrionuevo JS Baldo D (2009) A new species of *Telmatobius* (Anura, Ceratophryidae) from Northern Jujuy Province, Argentina. Zootaxa 2030: 1–20. 10.11646/zootaxa.2030.1.1

[B5] Benavides E Ortiz JC Formas JR (2002) A new species of *Telmatobius* (Anura: Leptodactylidae) from northern Chile. Herpetologica 58: 210–220. 10.1655/0018-0831(2002)058[0210:ANSOTA]2.0.CO;2

[B6] Berenguer J Cáceres I (2008) El Qhapaq Ñan en Chile. Tramo 2: Miño – Lasana, Región de Antofagasta. Informe presentado al Programa Qhapaq Ñan-Chile, Consejo de Monumentos Nacionales, Tagua Tagua Consultores, Santiago, Unpublished, 79 pp. 10.13140/RG.2.1.3931.4007

[B7] Blotto B Nuñez JJ Basso NG Úbeda CA Wheeler WC Faivovich J (2013) Phylogenetic relationships of a Patagonian frog radiation, the *Alsodes* + *Eupsophus* clade (Anura: Alsodidae), with comments on the supposed paraphyly of *Eupsophus*. Cladistics 29(2): 113–131. 10.1111/j.1096-0031.2012.00417.x 34814377

[B8] Capurro LF (1954) El género *Telmatobius* en Chile. Revista Chilena de Historia Natural 54(3): 31–40. https://rchn.biologiachile.cl/pdfs/1954-1955/3/Capurro_1954-1955.3.pdf

[B9] Capurro LF (1955) *Telmatobiushalliedentatus*. Nueva subespecie para la fauna anfibia de Chile. Investigaciones Zoológicas Chilenas 2: 150–152.

[B10] Cashins SD Alford RA Skerratt LF (2008) Lethal Effect of Latex, Nitrile, and Vinyl Gloves on Tadpoles. Herpetological Review 39(3): 298–301. https://researchonline.jcu.edu.au/6377/1/6377_Cashins_et_al_2008.pdf

[B11] Cei JM (1962) Batracios de Chile. Ediciones de la Universidad de Chile, Santiago, cviii + 128 pp.

[B12] Cei JM (1986) Speciation and adaptative radiation in Andean *Telmatobius* frogs. In: Vuilleumier F Monasterio M (Eds) High altitude tropical biogeography.Oxford University Press, New York, 374–386.

[B13] Collado G Vila I Méndez MA (2011) Monophyly, candidate species and vicariance in *Biomphalaria* snails (Mollusca: Planorbidae) from the Southern Andean Altiplano. Zoologica Scripta 40: 613–622. 10.1111/j.1463-6409.2011.00491.x

[B14] Correa C (2019) Nueva lista comentada de los anfibios de Chile (Amphibia, Anura). Boletín Chileno de Herpetología 6: 1–14. https://www.boletindeherpetologia.com/uploads/3/2/2/9/32291217/1._correa2019.pdf

[B15] Correa C (2021) A solution to the enigma of the type locality of *Telmatobiushalli* Noble, 1938 (Anura, Telmatobiidae), a frog lost for 86 years. ZooKeys 1060: 183–192. 10.3897/zookeys.1060.67904 34690508 PMC8486727

[B16] Correa C Durán F (2019) Taxonomy, systematics and geographic distribution of ground frogs (Alsodidae, *Eupsophus*): a comprehensive synthesis of the last six decades of research. ZooKeys 863: 107–152. 10.3897/zookeys.863.35484 31341395 PMC6639348

[B17] Csavina J Field J Taylor MP Gao S Landázuri A Betterton EA Sáez AE (2012) A review on the importance of metals and metalloids in atmospheric dust and aerosol from mining operations. Science of the Total Environment 433: 58–73. 10.1016/j.scitotenv.2012.06.013 PMC3418464 22766428

[B18] Cuevas CC Formas JR Alvarado-Rybak M Peñafiel-Ricaurte A Azat C (2020) Rediscovery of the enigmatic Andean frog *Telmatobiushalli* Noble (Anura: Telmatobiidae), re-description of the tadpole and comments on new adult’s characters, type locality and conservation status. Zootaxa 4834(2): 195–206. 10.11646/zootaxa.4834.2.2 33056120

[B19] De la Riva I (2005) Bolivian frogs of the genus *Telmatobius* (Anura: Leptodactylidae): synopsis, taxonomic comments, and description of a new species. In: Lavilla EO De la Riva I (Eds) Studies on the Andean Frogs of the Genera Telmatobius and Batrachophrynus.Asociacion Herpetológica Española, Monografías de Herpetología 7: 65–101.

[B20] De la Riva I García-París M Parra-Olea G (2010) Systematics of Bolivian frogs of the genus *Telmatobius* (Anura, Ceratophryidae) based on mtDNA sequences. Systematics and Biodiversity 8: 49–61. 10.1080/14772000903526454

[B21] Delsouc A Barber M Gallaud A Grings F Vidal-Páez P Pérez-Martínez W Briceño-De-Urbaneja I (2020) Seasonality Analysis of Sentinel-1 and ALOS-2/PALSAR-2 Backscattered Power over Salar de Aguas Calientes Sur, Chile. Remote Sensing 12(6): 941. 10.3390/rs12060941

[B22] Dill DB (1979) Case history of a physiologist: F. G. Hall. Physiologist 22(2): 8–21. https://citeseerx.ist.psu.edu/viewdoc/download?doi=10.1.1.462.2640&rep=rep1&type=pdf 377317

[B23] Dill DB (1980) Ten men on a mountain. In: Horvath SM Yousef MK (Eds) Environmental Physiology: Aging, Heat and Altitude.Elsevier, North Holland Inc., New York, 453–466.

[B24] Edgar RC (2004) MUSCLE: multiple sequence alignment with high accuracy and high throughput. Nucleic Acids Research 32(5): 1792–1797. 10.1093/nar/gkh340 15034147 PMC390337

[B25] Fabres A Fibla P Araya C Sallaberry M Méndez M (2018) Development and characterization of 22 polymorphic microsatellites of the Andean frog *Telmatobiuschusmisensis* (Anura, *Telmatobius*) and cross amplification in seven Chilean species of the genus. Molecular Biology Reports 45: 1533–1538. 10.1007/s11033-018-4228-2 29978382

[B26] Fibla P Sáez PA Salinas H Araya C Sallaberry M Méndez MA (2017) The taxonomic status of two *Telmatobius* (Anura: Telmatobiidae) frog species from the western Andean slopes of northernmost Chile. Zootaxa 4250(4): 301–314. 10.11646/zootaxa.4250.4.1 28610007

[B27] Fibla P Salinas H Lobos G Del Pozo T Fabres A Méndez MA (2018) Where is the enigmatic *Telmatobiushalli* Noble 1938? Rediscovery and clarification of a frog species not seen for 80 years. Zootaxa 4527(1): 61–74. 10.11646/zootaxa.4527.1.5 30651476

[B28] Flores CA (2001) Diagnóstico de la Cuenca Hidrográfica del Río Loa. Thesis, Universidad Católica del Norte, Antofagasta, Chile. http://bibliotecadigital.ciren.cl/bitstream/handle/123456789/13678/UCN-HUM0003.pdf

[B29] Formas JR Northland I Capetillo J Nuñez JJ Cuevas CC Brieva L (1999) *Telmatobiusdankoi*, una nueva especie de rana acuática del norte de Chile (Leptodactylidae). Revista Chilena de Historia Natural 72(3): 427–445. https://rchn.biologiachile.cl/pdfs/1999/3/Formas_et_al_1999.pdf

[B30] Formas JR Benavides E Cuevas C (2003) A new species of *Telmatobius* (Anura: Leptodactylidae) from Río Vilama, northern Chile, and the redescription of *T.halli* Noble. Herpetologica 59(2): 253–270. 10.1655/0018-0831(2003)059[0253:ANSOTA]2.0.CO;2

[B31] Formas JR Veloso A Ortiz JC (2005) Sinopsis de los *Telmatobius* de Chile. Monografías de Herpetología 7: 103–114.

[B32] Frost DR (2021) Amphibian Species of the World: an Online Reference. Version 6.1 (01/03/2021 8:15 a.m.). Electronic Database accessible at https://amphibiansoftheworld.amnh.org/index.php. American Museum of Natural History, New York. 10.5531/db.vz.0001

[B33] Gosner KL (1960) A simplified table for staging anuran embryos and larvae with notes on identification. Herpetologica 16(3): 183–190. https://www.jstor.org/stable/3890061?origin=JSTOR-pdf&seq=1

[B34] Hall T (1999) Bioedit: A user-friendly biological sequence alignment editor and analysis program for windows 95/98/NT. Version 5.0.9. Nucleic Acids Symposium Series 41: 95–98.

[B35] Hill A Prince P Snaddon J Doncaster C Rogers A (2019) AudioMoth: A low-cost acoustic device for monitoring biodiversity and the environment. HardwareX 6: 1–19. 10.1016/j.ohx.2019.e00073

[B36] Hoffman MA (1987) Ross A. McFarland Collection in Aerospace Medicine and Human Factors Engineering – 1 Catalog of the Library. Fordham Health Sciences Library, Wright State University School of Medicine. Dayton, Ohio, [x +] 158 pp. https://www.libraries.wright.edu/special/collectionguides/files/fsc1vol1.pdf

[B37] Hoffman MA Ritchie RA (1987) Ross A. McFarland Collection in Aerospace Medicine and Human Factors Engineering – 2 Inventory of the Manuscripts. Fordham Health Sciences Library, Wright State University School of Medicine. Dayton, Ohio, [xiv +] 182 pp. https://www.libraries.wright.edu/special/collectionguides/files/fsc1vol2.pdf

[B38] HUGIN – panorama photo stitcher (2020) HUGIN – panorama photo stitcher (version 2019.2.0). http://hugin.sourceforge.net [accessed 10/10/2020]

[B39] IUCN SSC Amphibian Specialist Group (2015) *Telmatobiushalli*. The IUCN Red List of Threatened Species, 2015, e.T21582A79809691. 10.2305/IUCN.UK.2015-4.RLTS.T21582A79809691.en

[B40] Johnson ML Speare R (2005) Possible modes of dissemination of the amphibian chytrid *Batrachochytriumdendrobatidis* in the environment. Diseases of Aquatic Organisms 65(3): 181–186. 10.3354/dao065181 16119886

[B41] Keys A (1936a) La vida en las grandes alturas. La expedición internacional de 1935 a Chile. Revista Geográfica Americana 3(35): 79–98.

[B42] Keys A (1936b) The physiology of life at high altitudes. The international high altitude expedition to Chile, 1935. The Scientific Monthly 43(4): 289–312. https://www.jstor.org/stable/16163

[B43] Keys A Matthews BHC Forbes WH McFarland RA (1938) Individual variations in ability to acclimatize to high altitude. Proceedings of the Royal Society of London B 126(842): 1–24. 10.1098/rspb.1938.0043

[B44] Lobos G Rojas O (2020) Ecología y Conservación en los *Telmatobius* Altoandinos de Chile; El Caso de la Ranita del Loa. Corporación de Cultura y Turismo de Calama, Calama, Chile, 169 pp. https://calamacultural.cl/bibliotecavirtual/pdf/Libro%20La%20Ranita%20del%20Loa%20.pdf

[B45] Lobos G Rebolledo N Sandoval M Canales C Perez-Quezada JF (2018) Temporal Gap Between Knowledge and Conservation Needs in High Andean Anurans: The Case of the Ascotán Salt Flat Frog in Chile (Anura: Telmatobiidae: *Telmatobius*). South American Journal of Herpetology 13(1): 33–43. 10.2994/SAJH-D-16-00062.1

[B46] Lobos G Sáez PA Villablanca R Prado M Cruz-Jofré F Fibla P Méndez MA (2020) Invasion of salmonids in the Puna and Southern Chilean Altiplano: patterns and threats to the biodiversity. BioInvasions Records 9(4): 853–864. 10.3391/bir.2020.9.4.19

[B47] McFarland R (1935) Ross McFarland’s Diary (May 1935–September 1935). Ross A. McFarland Collection in Aerospace Medicine and Human Factors Engineering, Wright State University Archives, Dayton, Ohio, 197 pp.

[B48] Mitchell MA (2009) Anesthetic considerations for amphibians. Journal of Exotic Pet Medicine 18(1): 40–49. 10.1053/j.jepm.2008.11.006

[B49] MMA: Ministerio de Medio Ambiente, Gobierno de Chile (2019) Ficha de antecedentes de especie: *Telmatobiushalli*. http://especies.mma.gob.cl/CNMWeb/Web/WebCiudadana/ficha_indepen.aspx?EspecieId=57&Version=1

[B50] Noble GK (1938) A new species of frog of the genus *Telmatobius* from Chile. American Museum Novitates 973: 1–3. http://digitallibrary.amnh.org/handle/2246/3914

[B51] Northland I Capetillo J Iturra P Veloso A (1990) Nuclear DNA content and karyosystematic relationships of species grouped in primitive tribes of Leptodactylidae (Amphibia-Anura). Revista Brasileira de Genetica 13: 247–254.

[B52] Núñez H Gálvez O (2015) Catálogo de la colección herpetológica del Museo Nacional de Historia Natural y nomenclátor basado en la colección. Publicación Ocasional del Museo Nacional de Historia Natural de Chile 64: 1–211. https://publicaciones.mnhn.gob.cl/668/articles-71131_archivo_01.pdf

[B53] Open Acoustic Devices (2020) AudioMoth Temperature Measurements. https://github.com/OpenAcousticDevices/Application-Notes/blob/master/AudioMoth_Temperature_Measurements.pdf

[B54] Padial JM Miralles A De la Riva I Vences M (2010) The integrative future of taxonomy. Frontiers in Zoology 7: e16. 10.1186/1742-9994-7-16 PMC2890416 20500846

[B55] Paicho-Hidalgo M Meza-Aliaga M Espinoza-González G Vera-Burgos C (2015) Impacto de eventos aluvionales sobre humedales de quebrada: el caso de Altuza e Iquiuca-Parca. Región de Tarapacá (Chile). Revista Geográfica del Sur 6(9): 1–17. http://www.revgeosur.udec.cl/wp-content/uploads/2016/10/Paicho-Hidalgo_et_al_2015_RGS.pdf

[B56] Rambaut A Drummond AJ Xie D Baele G Suchard MA (2018) Posterior Summarization in Bayesian Phylogenetics Using Tracer 1.7. Systematic Biology 67(5): 901–904. 10.1093/sysbio/syy032 29718447 PMC6101584

[B57] Reider KE Larson DJ Barnes BM Donnelly MA (2020) Thermal adaptations to extreme freeze-thaw cycles in the high tropical Andes. Biotropica 53(1): 296–306. 10.1111/btp.12875

[B58] Ronquist F Teslenko M van der Mark P Ayres DL Darling A Höhna S Larget B Liu L Suchard MA Huelsenbeck JP (2012) MrBayes 3.2: Efficient Bayesian Phylogenetic Inference and Model Choice Across a Large Model Space. Systematic Biology 61(3): 539–542. 10.1093/sysbio/sys029 22357727 PMC3329765

[B59] Ruiz G Rosenmann M Veloso A (1983) Respiratory and hematological adaptations to high altitude in *Telmatobius* frogs from the Chilean Andes. Comparative Biochemistry and Physiology Part A: Physiology 76(1): 109–113. 10.1016/0300-9629(83)90300-6

[B60] Sáez PA Fibla P Correa C Sallaberry M Salinas H Veloso A Mella J Iturra P Méndez MA (2014) A new endemic lineage of the Andean frog genus *Telmatobius* (Anura, Telmatobiidae) from the western slopes of the central Andes. Zoological Journal of the Linnean Society 171(4): 769–782. 10.1111/zoj.12152

[B61] Sáez PA Méndez MA (2020) Sistemática del Género *Telmatobius* en Chile. In: Lobos G Rojas O (Eds) Ecología y Conservación en los Telmatobius Altoandinos de Chile; El Caso de la Ranita del Loa.Corporación de Cultura y Turismo de Calama, Calama, 42–51. https://calamacultural.cl/bibliotecavirtual/pdf/Libro%20La%20Ranita%20del%20Loa%20.pdf

[B62] Solís R Penna M De la Riva I Fisher MC Bosch J (2015) Presence of *Batrachochytriumdendrobatidis* in anurans from the Andes highlands of northern Chile. Herpetological Journal 25(1): 55–59. http://repositorio.uchile.cl/handle/2250/167633

[B63] Soto-Azat C Valenzuela-Sánchez A Ortiz JC Díaz-Páez H Castro C Charrier A Correa C Cuevas C Lobos G Méndez MA Penna M Peñafiel-Ricaurte A Rabanal F Vélez-R CM Vidal MA Angulo A (2015) ASG Chile Leads Update of the Extinction Risk of Chilean Amphibians for The IUCN Red List of Threatened Species. FrogLog 23(4): 6–7. http://cis.unab.cl/wp-content/uploads/2017/03/Leads-Update-of-the-Extinction-Risk-of-Chilean-Amphibians-for-the-IUCN-Red-List-of-Threatened-SpeciesTM-FrogLog-2015-10-unab.pdf

[B64] Thomas V Van Rooij P Meerpoel C Stegen G Wauters J Vanhaecke L Martel A Pasmans F (2020) Instant killing of pathogenic chytrid fungi by disposable nitrile gloves prevents disease transmission between amphibians. PLoS ONE 15(10): 1–16. 10.1371/journal.pone.0241048 PMC7595420 33119670

[B65] Trueb L (1979) Leptodactylid frogs of the genus *Telmatobius* in Ecuador with the description of a new species. Copeia 4: 714–733. 10.2307/1443882

[B66] Veloso A Sallaberry M Navarro J Iturra P Valencia J Penna M Díaz N (1982) Contribución sistemática al conocimiento de la herpetofauna del extremo norte de Chile. In: Veloso A Bustos E (Eds) La Vegetación y vertebrados ectotérmicos del transecto Arica-Lago Chungará.ROSTLAC, Montevideo, 135–268.

[B67] Victoriano PF Muñoz-Mendoza C Sáez PA Salinas HF Muñoz-Ramírez C Sallaberry M Fibla P Méndez MA (2015) Evolution and Conservation on Top of the World: Phylogeography of the Marbled Water Frog (*Telmatobiusmarmoratus* Species Complex; Anura, Telmatobiidae) in Protected Areas of Chile. Journal of Heredity 106(S1): 546–559. 10.1093/jhered/esv039 26245789

[B68] Vila I Morales P Scott S Poulin E Véliz D Harrod C Méndez MA (2013) Phylogenetic and phylogeographic analysis of the genus *Orestias* (Teleostei: Cyprinodontidae) in the southern Chilean Altiplano: the relevance of ancient and recent divergence processes in the speciation. Journal of Fish Biology 82: 927–943. 10.1111/jfb.12031 23464552

[B69] Watters J Cummings S Flanagan R Siler C (2016) Review of morphometric measurements used in anuran species descriptions and recommendations for a standardized approach. Zootaxa 4072(4): 477–495. 10.11646/zootaxa.4072.4.6 27395941

[B70] Webb R Mendez D Berger L Speare R (2007) Additional disinfectants effective against the amphibian chytrid fungus *Batrachochytriumdendrobatidis*. Diseases of Aquatic Organisms 74(1): 13–6. 10.3354/dao074013 17425259

[B71] Wiens JJ (1993) Systematics of the leptodactylid frog genus *Telmatobius* in the Andes of northern Peru. Occasional Papers of the Museum of Natural History, The University of Kansas 162: 1–76.

[B72] Xia X (2018) DAMBE7: New and Improved Tools for Data Analysis in Molecular Biology and Evolution. Molecular Biology and Evolution 35(6): 1550–1552. 10.1093/molbev/msy073 29669107 PMC5967572

